# Indole Diketopiperazine Alkaloids Isolated From the Marine-Derived Fungus *Aspergillus chevalieri* MCCC M23426

**DOI:** 10.3389/fmicb.2022.950857

**Published:** 2022-07-07

**Authors:** Dongli Lv, Jinmei Xia, Xiaoqing Guan, Qiliang Lai, Beibei Zhang, Jianhui Lin, Zongze Shao, Sulan Luo, Dongting Zhangsun, Jiang-Jiang Qin, Weiyi Wang

**Affiliations:** ^1^Key Laboratory of Tropical Biological Resources of Ministry of Education, School of Pharmaceutical Sciences, Hainan University, Haikou, China; ^2^Key Laboratory of Marine Biogenetic Resources, Third Institute of Oceanography, Ministry of Natural Resources, Xiamen, China; ^3^The Cancer Hospital of the University of Chinese Academy of Sciences (Zhejiang Cancer Hospital), Institute of Basic Medicine and Cancer, Chinese Academy of Sciences, Hangzhou, China

**Keywords:** *Aspergillus*, cytotoxic, antibacterial, NMR, indole diketopiperazine

## Abstract

Two new indole diketopiperazines (**1–2**) obtained from the fermentation culture of a deep-sea-derived fungus *Aspergillus chevalieri* MCCC M23426, were characterized, together with nine biogenetic related compounds (**3–11**). The structures of **1–2** were assigned based on NMR, MS, NMR calculation, DP4+ analysis, and ECD calculation. The bioactive assay showed that compounds **1**, **5**–**7** significantly inhibited the growth of *Staphylococcus aureus*. Meanwhile, compound **8** potently reduced the cell viability of gastric cancer cell MKN1 with an IC_50_ value of 4.6 μM.

## Introduction

Indole diketopiperazines (indole DKPs) are a group of natural products with diketopiperazine backbones. The backbones are formed through the condensation of certain amino acids with L-tryptophan. There are multiple candidates for this amino acid, such as L-valine, L-tryptophan, L-alanine, or L-proline. After being modified by various tailoring enzymes the structures of the products can be very diverse. One family worth mentioning is the echinulin family which usually shared the same structure moiety of a cyclo-L-Trp-L-Ala with a prenyl group attached to C-2. The additional prenyl substituents and the diketopiperazine moiety with diverse oxidative states distinguish the members of this family from each other. Most of the reported echinulins were produced by fungi. Due to their diversity in structures and their broad biological and pharmacological activities, echinulins are of great interest to natural medicinal chemists. Several echinulins were reported to be promising leads. Neoechinulin A, isoechinulin A, and variecolorin G can scavenge free radicals such as DPPH. What is more, neoechinulin A also shows anti-inflammatory activity. It can function as a cytoprotective agent and can protect PC12 cells from peroxynitrite-induced death (Nies and Li, 2021).

Our previous work has shown that marine microorganisms are promising producers of bioactive compounds. The secondary metabolites of a deep-sea-derived *Aspergillus chevalieri* strain were studied in this work. The genus *Aspergillus* is very productive in bioactive compounds. Several bioactive products of *Aspergillus chevalieri* were reported. One new nonadride enantiomer named ent-epiheveadride was recently obtained from the marine-derived fungus *Aspergillus chevalieri* PSU-AMF79, together with five known dioxopiperazine derivatives (Ningsih et al., 2022).

In earlier research, sixteen metabolites were separated from the endolichenic fungus *Aspergillus chevalieri* SQ-8 (Lin et al., 2021). Seven of these compounds are C7-alkylated salicylaldehyde derivatives and nine are prenylated indole alkaloids. Interestingly, two indole DKPs were reported to be generated by the deep-sea cold-seep-derived fungus *Aspergillus chevalieri* CS-122 (Yan et al., 2022).

**FIGURE 1:**
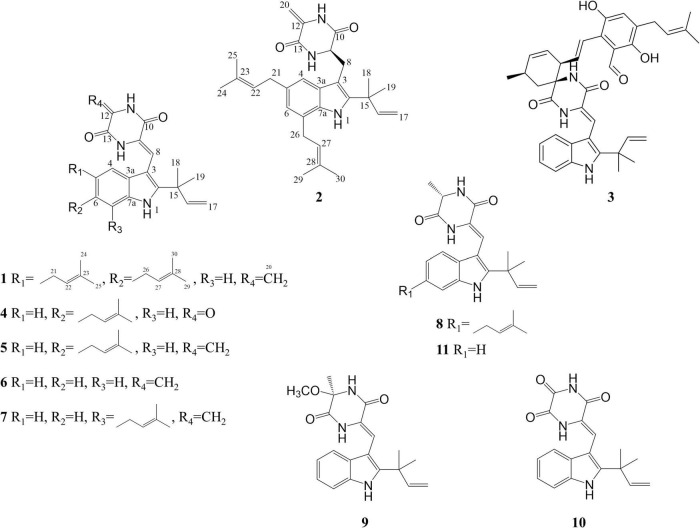
Structures of compounds **1**–**11**.

In this study, the extract of a deep-sea-derived *Aspergillus chevalieri* strain showed great potential in antibacterial and cytotoxic activities. Two new indole DKPs, **1–2**, were obtained from the extract by biological activity-tracking, together with nine biogenetic related compounds (**3–11**) (Figure 1). Their structures were determined by multiple spectroscopic methods and their bioactivities were also evaluated.

## Materials and Methods

### General Experimental Procedures

A Chirascan spectrometer (Applied Photophysics) was used to detect optical rotations of new compounds in MeOH. A UV-1800 spectrophotometer (Shimadzu, Kyoto, Japan) was used to collect UV (Ultra Violet) data for new compounds dissolved in MeOH. NMR spectra were collected on a Bruker Avance 850 MHz spectrometer. A Waters Xevo G2 Q-TOF mass spectrometer was utilized to obtain accurate molecular weight. MPLC (Medium Pressure Liquid Chromatography) was undertaken using different packing materials such as silica gel (200–300 mesh, 300–400 mesh, Tsingtao Marine Chemical Co., Ltd., Tsingtao, China), RP-C18 (ODS-A, 50 μm, YMC, Kyoto, Japan), and Sephadex LH-20 (GE Healthcare Bio-Science AB, Pittsburgh, PA, United States). HPLC (High-Performance Liquid Chromatography) was conducted on an Agilent 1260 system using an RP-C18 column (5 μm, 20 × 250 mm, YMC, Kyoto, Japan) with HPLC grade MeOH and MeCN as mobile phases.

### Fungal Material

Strain R10-1G1 of *Aspergillus chevalieri* was identified by ITS sequence homology [100% similarity with *Aspergillus chevalieri* NRRL 78 (TYPE material) with a Genbank accession No. NR_135340 (max score 955, e value 0.0, query cover 100%)]. A voucher specimen was deposited at the Marine Culture Collection of China (MCCC) with the preservation number MCCC M23426. The strain was streaked on potato dextrose agar (PDA) slants and stored at 4 °C until use.

### Fermentation, Extraction, and Isolation

Details about the fermentation, extraction, and isolation were provided in the supporting information (Supplementary Material, “Fermentation, Extraction, and Isolation”).

5-prenylcryptoechinulin A (**1**): white amorphous solid; UV λ_max_ (methanol) nm (log ε): 230 (4.71), 275 (4.45); ^1^H NMR and ^13^C NMR data are shown in Table 1; HR-ESI-MS: *m*/*z* 456.2650 [M – H]^–^ (Calcd. 456.2651 for C_29_H_34_N_3_O_2_, Δ – 0.2 ppm).

**TABLE 1:** ^1^H NMR data (850 MHz) and ^13^C NMR data (214 MHz) for compounds **1–3** (chloroform-*d*).

No.	1		2		3	
	δ_C_, type	δ_H_ Mult. (*J* in Hz)	δ_C_, type	δ_H_ Mult. (*J* in Hz)	δ_C_, type	δ_H_ Mult. (*J* in Hz)
1	NH	8.18 (s, 1H)	NH	8.07 (s, 1H)	NH	8.26 (s, 1H)
2	143.9, C		141.6, C		144.0, C	
3	102.8, C		103.6, C		102.9, C	
4	118.8, CH	7.07 (s, 1H)	115.0, CH	7.14 (d, 1.5 Hz, 1H)	118.7, CH	7.12 (dd, 8.0,1.6 Hz, 1H)
5	133.5, C		134.0, C		121.2, CH	7.10 (ddd, 7.9,6.6,0.9 Hz, 1H)
6	134.9, C		122.9, CH	6.81 (d, 1.5 Hz, 1H)	122.5, CH	7.19 (dd, 6.6,1.6 Hz, 1H)
7	111.1, CH	7.17 (s, 1H)	123.5, C		111.3, CH	7.34 (m, 1H)
8	113.6, CH	7.28 (s, 1H)	30.7, CH_2_	3.63 (dd, 14.5,3.8 Hz, 1H)	112.6, CH	7.18 (s, 1H)
8			30.7, CH_2_	3.26 (dd, 14.5,12.0 Hz, 1H)		
9	123.7, C		55.6, CH	4.54 (ddd, 12.0,3.9,2.0 Hz, 1H)	124.2, C	
10	157.7, C		166.3, C		167.2, C	
11	NH	7.68 (s, 1H)	NH		NH	7.39 (s, 1H)
12	133.6, C		133.0, C		60.5, C	
13	155.5, C		158.0, C		161.8, C	
14	NH	8.29 (s, 1H)	NH	5.75 (s, 1H)	NH	6.36 (s, 1H)
15	39.3, C		38.9, C		39.1, C	
16	144.3, CH	6.05 (dd, 17.5,10.5 Hz, 1H)	145.7, CH	6.08 (dd, 17.4,10.5 Hz, 1H)	144.1, CH	6.00 (dd, 17.4,10.5 Hz, 1H)
17	113.3, CH_2_	5.21 (dd, 10.5,1.0 Hz, 1H)	112.2, CH_2_	5.15 (dd, 10.5,1.0 Hz, 1H)	113.5, CH_2_	5.19 (dd, 10.5,1.0 Hz, 1H)
17	113.3, CH_2_	5.17 (dd, 17.5,1.0 Hz, 1H)		5.13 (dd, 17.1,1.0 Hz, 1H)	113.5, CH_2_	5.13 (dd, 17.5,1.0 Hz, 1H)
18	27.5, CH_3_	1.51 (s, 3H)	27.7, CH_3_	1.50 (s, 3H)	27.3, CH_3_	1.40 (d, 4.8 Hz, 3H)
19	27.5, CH_3_	1.51 (s, 3H)	27.8, CH_3_	1.49 (s, 3H)	27.3, CH_3_	1.40 (d, 4.8 Hz, 3H)
20	101.6, CH_2_	5.60 (d, 1.2 Hz, 1H)	102.0, CH_2_	5.59 (d, 1.2 Hz, 1H)	116.8, C	
20	101.6, CH_2_	4.94 (d, 1.2 Hz, 1H)	102.0, CH_2_	4.88 (d, 1.2 Hz, 1H)		
21	31.9, CH_2_	3.39 (m, 2H)	34.6, CH_2_	3.39 (m, 2H)	155.5, C	
22	123.5, CH	5.27 (m, 1H)	124.5, CH	5.35 (m, 1H)	131.0, C	
23	132.4, C		131.6, C		126.8, CH	7.02 (s, 1H)
24	17.8, CH_3_	1.69 (s, 3H)	25.8, CH_3_	1.74 (s, 3H)	145.5, C	
25	25.6, CH_3_	1.72 (s, 3H)	17.9, CH_3_	1.75 (s, 3H)	124.3, C	
26	31.7, CH_2_	3.40 (m, 2H)	31.4, CH_2_	3.54 (m, 2H)	126.7, CH	6.75 (dd, 16.2,0.9 Hz, 1H)
27	123.1, CH	5.31 (m, 1H)	122.9, CH	5.43 (m, 1H)	137.5, CH	5.89 (dd, 16.2,8.0 Hz, 1H)
28	132.6, C		133.0, C		46.1, CH	3.70 (dtd, 7.7,3.3,2.3 Hz, 1H)
29	25.8, CH_3_	1.78 (s, 3H)	17.9, CH_3_	1.87 (s, 3H)	124.1, CH	5.73 (dt, 10.1,2.6 Hz, 1H)
30	17.9, CH_3_	1.71 (s, 3H)	25.7, CH_3_	1.81 (s, 3H)	134.4, CH	5.96 (dt, 10.2,2.9 Hz, 1H)
31					27.9, CH	2.71 (dq, 10.9,3.2 Hz, 1H)
32					38.6, CH_2_	2.52 (dd, 13.9,7.9 Hz, 1H)
32					38.6, CH_2_	1.86 (dd, 13.9,3.5 Hz, 1H)
33					22.0, CH_3_	1.26 (d, 7.5 Hz, 3H)
34					196.0, CH	10.06 (s, 1H)
35					27.1, CH_2_	3.30 (d, 7.4 Hz, 1H)
36					120.9, CH	5.27 (dddd, 7.4,6.0,2.9,1.4 Hz, 1H)
37					133.9, C	
38					17.8, CH_3_	1.68 (m, 3H)
39					25.8, CH_3_	1.74 (d, 1.3 Hz, 3H)
7a	133.3, C		132.2, C		134.2, C	
3a	124.1, C		128.8, C		126.0, C	
OH-21					OH	11.80 (s, 1H)

9-*epi*-didehydroechinulin (**2**): white amorphous solid; [α]D25.0-98.9⁢(c⁢ 0.1,MeOH);UV λ_max_ (methanol) nm (log ε): 230 (4.56), 275 (4.14); ^1^H NMR and ^13^C NMR data are shown in Table 1; HR-ESI-MS: *m*/*z* 482.2784 [M + Na]^+^ (Calcd. 482.2783 for C_29_H_37_N_3_O_2_Na, Δ + 0.2 ppm).

### NMR Calculation

The conformer rotamer ensemble sampling tool (crest) (Pracht et al., 2020) was utilized to generate candidate conformers and DFT calculations were performed using the Gaussian 16 program (Frisch et al., 2016). The shielding constants were calculated by the GIAO method with TMS (Willoughby et al., 2014). More details about the computations and DP4+ analysis were provided in the supporting information (Supplementary Material, “NMR Calculation”) (Wang et al., 2018a,2020a,2020b).

### Antitumor and Antibacterial Assay

The human gastric cancer cell line MKN1 was purchased from American Type Culture Collection (ATCC, LGC Standards SLU, Barcelona, Spain). The *in vitro* assay was carried out to investigate the effects of compounds **1-11** on the gastric cancer cells. The cell viability (CCK8 assays), colony formation, and cell apoptosis (Annexin V-FITC Apoptosis Staining/Detection Kit, BD, United States) were all evaluated and the details were described previously (Wang et al., 2014, 2018c,2019a). The protocol for antibacterial assay has also been described previously (Wang et al., 2018a,b, 2019b).

## Results

**FIGURE 2:**
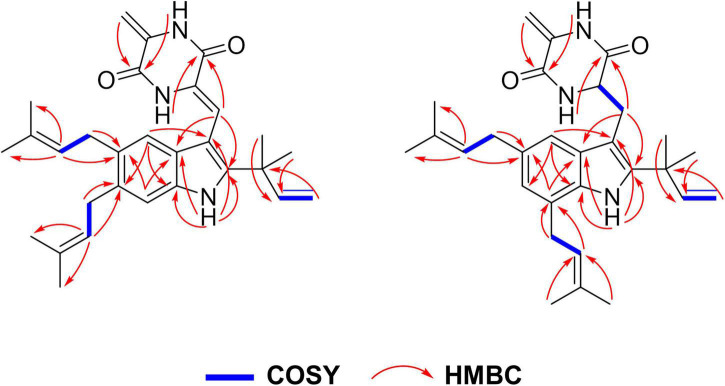
Key COSY and HMBC correlations of compounds **1–2**.

Compound **1** was isolated in the form of a colorless amorphous solid. Inspection of the HR-ESI-MS at *m/z* 456.2650 [M – H]^–^ (calcd. 456.2651 for C_29_H_34_N_3_O_2_, Δ – 0.2 ppm, Supplementary Figure 1) allowed the establishment of its molecular formula as C_29_H_35_N_3_O_2_ with 14 degrees of unsaturation. The HSQC and ^13^C/DEPT135 spectra evidenced six methyl groups (C-18, 19, 24, 25, 29, and 30), five methylene groups (C-17, 20, 21, and 26), six methine groups (C-4, 7, 8, 16, 22, and 27), and ten quaternary carbons (C-2, 3, 5, 6, 3a, 7a, 9, 10, 12, 13, 15, 23, and 28) (Supplementary Figures 2, 3). Interpretation of UV absorptions at λ_max_ 230 and 275 nm delineated the amide and conjugated indole moieties in **1** (Supplementay Figure 4). Cross peaks of H-4/C-6 and 7a, NH/C-2, 3, 3a and 7a, and H-7/C-5 and 3a, constructed the indole moiety. Cross peaks of H-8/C-2 and 3a (Figure 2 and Supplementary Figure 5) in HMBC spectra placed the C-8–C-9–C-10 unit at C-3. Detailed inspection of NMR data of **1** proved that **1** might be an indole diketopiperazine, structurally close to cryptoechinulin A (Supplementary Figures 6, 7). Additional signals were deduced to be a prenyl group connecting to C-5, based on the apparent single peaks at δ_H_ 7.07 and 7.17 (coupling constants were small and negligible). This was also supported by the HMBC correlation of H-22/C-5 (Table 1 and Supplementary Figure 8). As the NMR data was insufficient to support the configuration of C-8, NMR calculations at MPW1PW91-SCRF/6–31+G(d,p)//B3LYP/6-31G(d) for two candidate isomers (8*Z*)-**1** and (8*E*)-**1** were carried out. Through the comparison of the key computation parameters, as shown in Table 2, the calculated data of (8*Z*)-**1** matched well with their experimental counterparts. In addition, the 100% DP4+ probability of (8*Z*)-**1** (Table 2, Supplementary Tables 1–15, and Supplementary Figure 9) also supported this deduction. The structure of **1** was established and named 5-prenylcryptoechinulin A.

**TABLE 2:** Comparison of the key parameters of (8*Z*)-**1**, (8*E*)-**1,** and **3a–3d** in NMR computations.

	(8*Z*)-1	(8*E*)-1	3a	3b	3c	3d
*R*^2^ (^13^C)	0.9971	0.9962	0.9969	0.9956	0.9989	0.9985
MAE (^13^C)	2.4	2.8	3.3	2.6	1.5	1.7
CMAE (^13^C)	2	2.4	1.9	2.6	1.4	1.7
*R*^2^ (^1^H)	0.9953	0.9918	0.9454	0.9619	0.9954	0.9847
MAE (^1^H)	0.15	0.14	0.57	0.57	0.3	0.39
CMAE (^1^H)	0.1	0.13	0.3	0.28	0.1	0.19
DP4+ (^13^C)	100.00%	0.00%	NA	NA	NA	NA
DP4+ (^1^H)	100.00%	0.00%	NA	NA	NA	NA
DP4+ (all data)	100.00%	0.00%	NA	NA	NA	NA

**FIGURE 3:**
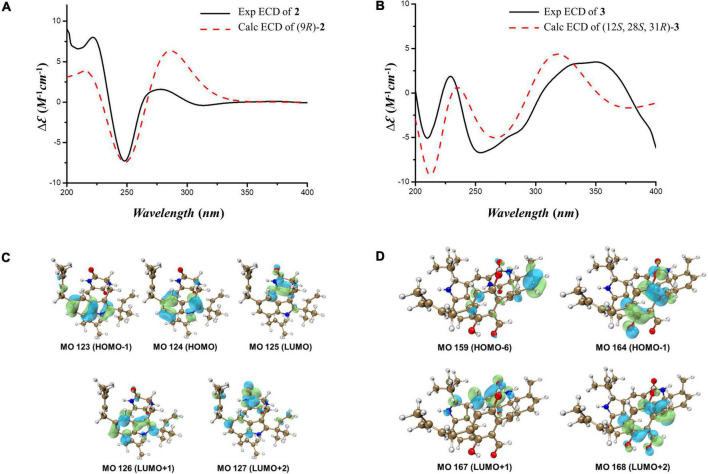
**(A,B)** Calculated and experimental ECD spectra of **2** and **3**. **(C,D)** Key molecular orbitals (MOs) involved in the important transitions of **2** and **3**.

Compound **2** was isolated as a colorless amorphous solid. The UV spectra of **1** and **2** were similar which demonstrated that they shared the same backbone (Supplementary Figure 10). The molecular formula C_29_H_37_N_3_O_2_ with 13 degrees of unsaturation (Supplementary Figure 11) indicated that one double bond was absent in **2** compared to **1,** and **2** was a hydrogenated derivative of **1**. The correlations of H_2_-8 to C-2 and C-3a and of H-9 to C-3, C-10 and C-13 in the HMBC spectrum allowed the elucidation of the hydrogenated bond C-8-C-9 (Figure 2 and Supplementary Figures 12–16). The ^1^H-NMR signals at δ 7.14 (d, *J* = 1.5 Hz) and 6.81 (d, *J* = 1.5 Hz) indicated the presence of a 4,6 or 5,7 disubstituted benzene moiety (Table 1 and Supplementary Figure 17). The HMBC correlation of H-4/C-3 further placed the two prenyls at C-5 and C-7, respectively (Figure 2 and Supplementary Figure 15), excluding the possibility of 4,6-disubstitution. Next, TDDFT calculation of the ECD spectrum at Cam-B3LYP/Def2SVP (Supplementary Tables 16–22) revealed the stereochemistry of C-9 as *R* (Figure 3A), as the theoretical ECD curve was in agreement with the experimental one. The molecular orbital (MO) analysis was carried out for the optimal conformer of **2a** (65.2%). The positive Cotton effect (CE) at ∼282 nm of **2** was thus related to electron transition from MO124 (HOMO) to MO125 (LUMO). The negative CE at ∼251 nm could be ascribed to the electron transition from MO124 (HOMO) to MO126 (LUMO+1). The positive CE at ∼219 nm was caused by the electron transition from MO124 (HOMO) to MO127 (LUMO+2) (Figure 3C). Finally, compound **2** was assigned as 9-*epi*-didehydroechinulin.

**FIGURE 4:**
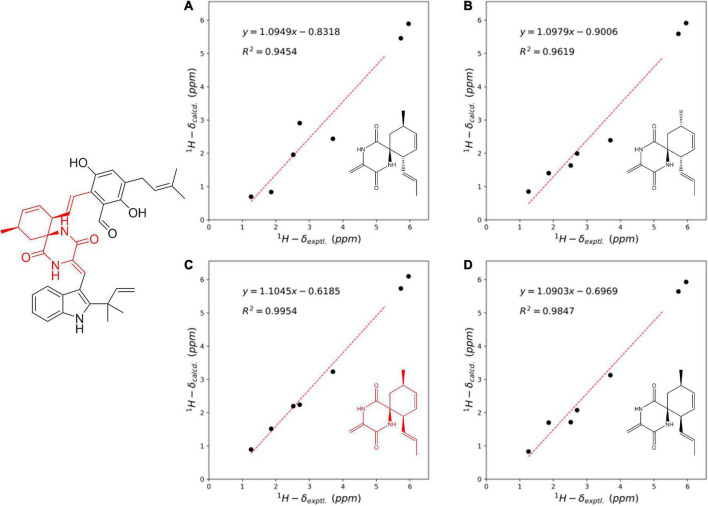
Linear regression analysis between the experimental and calculated NMR data of simplified conformers of **3a–d** at C-12, 13, 28, 29, 30, 31, 32, 33.

Compound **3** was obtained as a colorless amorphous solid. Detailed analysis of the NMR (Supplementary Figures 18–23) and HR-ESI-MS data revealed that **3** shared the same planar structure as the known compound cryptoechinuline D, which was firstly isolated from *Aspergillus amstelodami* in 1976 (Gatti et al., 1976). The relative stereochemistry of cryptoechinuline D was previously determined to be 12S*, 28R*, and 31R* (Gao et al., 2013). In their work, two epimers of cryptoechinuline D were obtained by chiral HPLC and assigned by CD spectra as (12*R*, 28*S*, 31*S*) and (12*S*, 28*R*, 31*R*), respectively. However, after carefully examining the stereochemistry of C-12, 28, and 31 of cryptoechinuline D, we found that the relative stereochemistry reported by Gao (Gao et al., 2013) was wrong and actually should be assigned as 12S*, 28S*, 31R*. To unambiguously establish the relative configuration of compound **3** in the current study, we simplified the candidate structures for DFT calculations as **3a–d** (Figure 4) and carried out the NMR calculation. By comparison of the calculated ^1^H NMR and ^13^C NMR of C-12, 13, 28, 29, 30, 31, 32, 33, the calculated **3c**, corresponding to (12S*, 28S*, 31R*)-cryptoechinuline D, was found to be in better agreement with the experimental data, as indicated by the correlation coefficient (R^2^), the mean absolute error (MAE), and the corrected mean absolute error (CMAE) (Table 2). Subsequently, TDDFT ECD calculation at the CAM-B3LYP-SCRF/def2-SVP (IEFPCM) level of theory was performed to elucidate the absolute configuration of compound **3** as 12*S*, 28*S*, 31*R* (Figure 3B and Supplementary Tables 23–27). The MO analysis of the optimal conformer of (12*S*, 28*S*, 31*R*)-cryptoechinuline D (35.9%) revealed that the negative CE at ∼250 nm in the experimental curve of **3** was related to the electron transition from MO164 (HOMO-1) to MO168 (LUMO+2). The negative CE at ∼206 nm could be ascribed to the electron transition from MO159 (HOMO-6) to MO167 (LUMO+1) (Figure 3D). Finally, compound **3** was assigned as (12*S*, 28*S*, 31*R*)-cryptoechinulin D.

The known compounds (**4**–**11**) were identified as neoechinuline (**4**) (Barbetta et al., 1969), cryptoechinulin A (**5**) (Cardillo et al., 1974), neoechinulin B (**6**) (Dossena et al., 1974), 7-prenylneoechinulin B (**7**) (Glisic et al., 2015; Nies and Li, 2021), neoechinulin D (**8**) (Dossena et al., 1975), variecolorin H (**9**) (Wang et al., 2007), cryptoechinulin C (**10**) (Cardillo et al., 1975), and neoechinulin A (**11**) (Cardillo et al., 1975), respectively, by comparing their spectroscopic data with those reported in the literature.

We first evaluated the antibacterial activities of **1**–**11** using strains of *Staphylococcus aureus* CICC 10384, *Vibrio parahaemolyticus* VP-HL, *Vibrio parahaemolyticus* ATCC 17802, and *Escherichia coli* CICC 10302. At the concentration of 250 μM, compounds **1**, **5**–**7** displayed selective antibacterial activities against *Staphylococcus aureus* CICC 10384 with an inhibition rate of over 90%, while compounds **8** and **10** exhibited moderate and weak antibacterial activities with an inhibition rate of 76 and 41%, respectively. Compound **7** showed potent antibacterial activity against *Staphylococcus aureus* CICC 10384 with a MIC value of 62.5 μM (positive control: chloramphenicol, MIC: 15.5 μM). We then evaluated the cytotoxicity of **1**–**11** against the gastric cancer cell line MKN1. Compounds **6** and **8** exhibited growth inhibition effects with IC_50_ values of 20.7 and 4.6 μM, respectively. Other compounds displayed weak cytotoxic activities with IC_50_ values over 100 μM (positive control: cisplatin, IC_50_: 8.8 μM).

## Discussion

Indole DKPs are an important group of bioactive metabolites usually derived from fungi (Ma et al., 2016). *Aspergillus* is the most important genera in producing indole DKPs with great diversity in structures. Seven new prenylated indole DKPs were isolated from *Aspergillus fumigatus* (Wang et al., 2008). Three new indole DKPs were obtained from the *Aspergillus taichungensis* ZHN-7-07 (Cai et al., 2015). Three new indole DKPs were reported to be produced by *Aspergillus ochraceus* (Wen et al., 2018). A pair of enantiomeric indole DKPs were isolated from the mangrove endophytic fungus *Aspergillus* sp. SK-28 (Cai et al., 2019). Three new indole DKPs were generated by a soft coral-associated epiphytic fungus *Aspergillus* sp. EGF 15-0-3 (Wei et al., 2020). New indole DKPs were also obtained from the mangrove rhizosphere soil-derived fungus, *Aspergillus effuses* H1-1 (Gao et al., 2013), the terrestrial-derived endophytic fungus *Aspergillus* sp. (Lhamo et al., 2015), the soil-derived fungus *Aspergillus ochraceopetaliformis* (Mostafa et al., 2021), the marine-derived fungus *Aspergillus* sp. Z3 (Li et al., 2022), the marine endophytic fungus *Aspergillus* sp. YJ191021 (Yang et al., 2021), the deep-sea-derived fungus *Aspergillus* sp. FS445 (Liu et al., 2021), and so on. In this work, two new indole DKPs were purified from the fermentation of the *Aspergillus chevalieri* MCCC M23426. This further demonstrated that the capacity of the genera *Aspergillus* in producing novel indole DKPs is yet to be fully explored.

In addition to their structural diversity, indole DKPs also exhibit a variety of activities (Jia et al., 2018). The cytotoxic activities of different indole DKPs on MOLT-4, A549, HL-60 (Cai et al., 2015), and BEL-7420 cell lines (Wang et al., 2008), on P388, HL-60, BEL-7402, and A-549 cell lines (Gao et al., 2013), on the prostate cancer PC3 cell line (Li et al., 2022), as well as on the NCI-H1975 gefitinib resistance (NCI-H1975/GR) cell lines (Wei et al., 2020) were evaluated. An indole diketopiperazine named asperthrins A was reported to possess antifungal and antibacterial activities against *Vibrio anguillarum*, *Xanthomonas oryzae* pv. *Oryzicola*, and *Rhizoctonia solani* (Yang et al., 2021). Another indole diketopiperazine named penilline D was assayed for its cytotoxic, antibacterial, and enzyme inhibitory activities against acetylcholinesterase (AChE) and pancreatic lipase (PL) but no significant activity was observed (Hu et al., 2021). Other reported activity assessments include anti-inflammatory effects (Wen et al., 2018), NO production inhibitory activity (Liu et al., 2021), radical scavenging activity against DPPH radicals (Zou et al., 2014), plant growth regulation (Zhang et al., 2013), and antifouling activity against the barnacle *Balanus reticulatus* (Cai et al., 2019). In this study, the inhibition effects of the new indole DKPs on several bacterial strains including the *Staphylococcus aureus*, and their cytotoxic activity against the gastric cancer cell MKN1, were evaluated. Although a variety of models have been applied to evaluate the bioactivities of indole DKPs, the activities observed were usually not significant. In-depth structure-activity relationship studies of these compounds, as well as structural modifications, may bring new chances for finding new structures with better bioactivities.

The cooccurrence of the echinulin family alkaloids also indicated some clues about their biosynthesis. Recent research heterologously expressed the putative echinulin biosynthetic gene cluster from *Aspergillus ruber* in *Aspergillus nidulans* (Nies and Li, 2021). Their work proved that EchPT2 catalyzes the prenylation steps and EchP450 catalyzes the formation of the double bond between C10 and C11. Compound **1** is the prenylated product of compound **5** at C5 whereas compound **7** is the prenylated product of compound **6** at C7. It is possible that these prenylation steps are also catalyzed by EchPT2. It was reported that preechinulin can be converted by the cytochrome P450 enzyme EchP450 to form neoechinulin A with a double bond and it can also be converted by EchPT2 to form prenylated products (Nies and Li, 2021). Here in this work, the obtained compounds **2** and **6** were closely related to a previously reported compound dihydroneochinulin B (Gao et al., 2013). The difference between compound **6** and dihydroneochinulin B lies in the double bond between C8 and C9. Compound **2** can be taken as the bioconversion product of dihydroneochinulin B after two prenylation steps at C5 and C7. It is possible that dihydroneochinulin B can be converted either by EchP450 to form compound **6** or by EchPT2 to produce compound **2**.

## Conclusion

In the current research, we have isolated two new indole DKPs (**1**–**2**) and nine biogenetically related compounds (**3**–**11**) from the deep-sea-derived *Aspergillus chevalieri* strain. Their structures were elucidated by extensive spectroscopic methods, NMR, and ECD calculations. Compounds **1**, and **5**–**7** selectively inhibited the growth of *Staphylococcus aureus* at the concentration of 250 μM. Meanwhile, compound **8** potently reduced the cell viability of gastric cancer cell MKN1 with an IC_50_ value of 4.6 μM. The present study demonstrates that the fungal indole DKPs are promising candidates for the discovery of new lead compounds for antimicrobial and anticancer therapy.

## Data Availability Statement

The datasets presented in this study can be found in online repositories. The names of the repository/repositories and accession number(s) can be found in the article/Supplementary Material.

## Author Contributions

DL, JX, XG, JL, and WW: investigation. QL and ZS: resources. JX, BZ, and WW: data analysis. JX, WW, and J-JQ: writing—original draft preparation. ZS, SL, and DZ: writing-revision. All authors have read and agreed to the published version of the manuscript.

## Conflict of Interest

The authors declare that the research was conducted in the absence of any commercial or financial relationships that could be construed as a potential conflict of interest.

## Publisher’s Note

All claims expressed in this article are solely those of the authors and do not necessarily represent those of their affiliated organizations, or those of the publisher, the editors and the reviewers. Any product that may be evaluated in this article, or claim that may be made by its manufacturer, is not guaranteed or endorsed by the publisher.

## References

[B1] Barbetta M. Casnati G. Pochini A. Selva A. (1969). Neoechinuline: a new indole metabolite from aspergillus amstelodami. *Tetrahedron Lett.* 10 4457–4460. 10.1016/S0040-4039(01)88723-2

[B2] Cai R. Jiang H. Xiao Z. Cao W. Yan T. Liu Z. (2019). (–)- and (+)-asperginulin A, a pair of indole diketopiperazine alkaloid dimers with a 6/5/4/5/6 pentacyclic skeleton from the mangrove endophytic fungus *Aspergillus* sp. SK-28. *Org. Lett.* 21 9633–9636. 10.1021/acs.orglett.9b03796 31762277

[B3] Cai S. Sun S. Peng J. Kong X. Zhou H. Zhu T. (2015). Okaramines S-U, three new indole diketopiperazine alkaloids from *Aspergillus taichungensis* ZHN-7-07. *Tetrahedron* 71 3715–3719. 10.1016/j.tet.2014.09.019

[B4] Cardillo R. Fuganti C. Gatti G. Ghiringhelli D. Grasselli P. (1974). Molecular structure of cryptoechinuline A, a new metabolite of *Aspergillus amstelodami*, isolated during investigations on echinuline biosynthesis. *Tetrahedron Lett.* 15 3163–3166. 10.1016/S0040-4039(01)91850-7

[B5] Cardillo R. Fuganti C. Ghiringhelli D. Grasselli P. Gatti G. (1975). Stereochemical course of the α,β-desaturation of L-tryptophan in the biosynthesis of cryptoechinuline A in *Aspergillus amstelodami*. *J. Chem. Soc. Chem. Commun.* 4 778–779. 10.1039/C39750000778

[B6] Dossena A. Marchelli R. Pochini A. (1974). New metabolites of *Aspergillus amstelodami* related to the biogenesis of neoechinulin. *J. Chem. Soc. Chem. Commun.* 3 771–772. 10.1039/c39740000771 558214

[B7] Dossena A. Marchelli R. Pochini A. (1975). Neoechinulin D, a new isoprenylated dehydrotryptophyl metabolite from *Aspergillus amstelodami*. *Experientia* 31 1249.

[B8] Frisch M. J. Trucks G. W. Schlegel H. B. Scuseria G. E. Robb M. A. Cheeseman J. R. (2016). *Gaussian 16 Rev*. *C.01.* Wallingford, CT.

[B9] Gao H. Zhu T. Li D. Gu Q. Liu W. (2013). Prenylated indole diketopiperazine alkaloids from a mangrove rhizosphere soil-derived fungus *Aspergillus effuses* H1-1. *Arch. Pharm. Res.* 36 952–956. 10.1007/s12272-013-0107-5 23539310

[B10] Gatti G. Cardillo R. Fuganti C. Ghiringhelli D. (1976). Structure determination of two extractives from *Aspergillus amstelodami* by nuclear magnetic resonance spectroscopy. *J. Chem. Soc. Chem. Commun.* 5 435–436.

[B11] Glisic S. Veljkovic N. Stanojevic M. Gemovic B. Perovic V. Radosevic D. (2015). Natural products as promising therapeutics for treatment of influenza disease. *Curr. Pharm. Des.* 21 5573–5588. 10.2174/1381612821666151002113426 26429712

[B12] Hu Y.-W. Chen W.-H. Song M.-M. Pang X.-Y. Tian X.-P. Wang F.-Z. (2021). Indole diketopiperazine alkaloids and aromatic polyketides from the Antarctic fungus *Penicillium* sp. SCSIO 05705. *Nat. Prod. Res.* 10.1080/14786419.2021.1973460 [Epub ahead of print]. 34498972

[B13] Jia B. Ma Y. Chen D. Chen P. Hu Y. (2018). Studies on structure and biological activity of indole diketopiperazine alkaloids. *Prog. Chem.* 30 1067–1081. 10.7536/PC171231

[B14] Lhamo S. Wang X.-B. Li T.-X. Wang Y. Li Z.-R. Shi Y.-M. (2015). Three unusual indole diketopiperazine alkaloids from a terrestrial-derived endophytic fungus, *Aspergillus* sp. *Tetrahedron Lett.* 56 2823–2826. 10.1016/j.tetlet.2015.04.058

[B15] Li X. Xu J. Wang P. Ding W. (2022). Novel indole diketopiperazine stereoisomers from a marine-derived fungus *Aspergillus* sp. Mycology-Intern. *J. Fung. Bio.* 10.1080/21501203.2022.2069173 [Epub ahead of print]. PMC9930829 36816774

[B16] Lin L.-B. Gao Y.-Q. Han R. Xiao J. Wang Y.-M. Zhang Q. (2021). Alkylated salicylaldehydes and prenylated indole alkaloids from the endolichenic fungus *Aspergillus chevalieri* and their bioactivities. *J. Agric. Food Chem.* 69 6524–6534. 10.1021/acs.jafc.1c01148 34096711

[B17] Liu Z. Chen Y. Li S. Hu C. Liu H. Zhang W. (2021). Indole diketopiperazine alkaloids from the deep-sea-derived fungus *Aspergillus* sp. FS445. *Nat. Prod. Res.* 10.1080/14786419.2021.1925271 [Epub ahead of print]. 33977842

[B18] Ma Y. M. Liang X. A. Kong Y. Jia B. (2016). Structural diversity and biological activities of indole diketopiperazine alkaloids from fungi. *J. Agric. Food Chem.* 64 6659–6671. 10.1021/acs.jafc.6b01772 27538469

[B19] Mostafa A. A. Dennis A. Ahmed A.-R. S. Ioanna C. Nikolas F. Mohamed S. (2021). New indole diketopiperazine alkaloids from a soil-derived fungus *Aspergillus ochraceopetaliformis*. *Planta Med.* 87 1316–1317. 10.1055/s-0041-1736992

[B20] Nies J. Li S. M. (2021). Prenylation and dehydrogenation of a C2-reversely prenylated diketopiperazine as a branching point in the biosynthesis of echinulin family alkaloids in *Aspergillus ruber*. *ACS Chem. Biol.* 16 185–192. 10.1021/acschembio.0c00874 33381959

[B21] Ningsih B. N. S. Rukachaisirikul V. Phongpaichit S. Preedanon S. Sakayaroj J. Muanprasat C. (2022). A nonadride derivative from the marine-derived fungus *Aspergillus chevalieri* PSU-AMF79. *Nat. Prod. Res.* 10.1080/14786419.2022.2039651 [Epub ahead of print]. 35168452

[B22] Pracht P. Bohle F. Grimme S. (2020). Automated exploration of the low-energy chemical space with fast quantum chemical methods. *Phys. Chem. Chem. Phys.* 22 7169–7192. 10.1039/c9cp06869d 32073075

[B23] Wang F. Fang Y. Zhu T. Zhang M. Lin A. Gu Q. (2008). Seven new prenylated indole diketopiperazine alkaloids from holothurian-derived fungus *Aspergillus fumigatus*. *Tetrahedron* 64 7986–7991. 10.1016/j.tet.2008.06.013

[B24] Wang W. Chen R. Luo Z. Wang W. Chen J. (2018a). Antimicrobial activity and molecular docking studies of a novel anthraquinone from a marine-derived fungus *Aspergillus versicolor*. *Nat. Prod. Res.* 32 558–563. 10.1080/14786419.2017.1329732 28511613

[B25] Wang W. Cheng J. W. Qin J. J. Hu B. Li X. Nijampatnam B. (2019a). MDM2-NFAT1 dual inhibitor, MA242: effective against hepatocellular carcinoma, independent of p53. *Cancer Lett.* 459 156–167. 10.1016/j.canlet.2019.114429 31181320 PMC6650270

[B26] Wang W. Liao Y. Chen R. Hou Y. Ke W. Zhang B. (2018b). Chlorinated azaphilone pigments with antimicrobial and cytotoxic activities isolated from the deep sea derived fungus *Chaetomium* sp. NA-S01-R1. *Mar. Drugs* 16:61. 10.3390/md16020061 29438326 PMC5852489

[B27] Wang W. Liao Y. Zhang B. Gao M. Ke W. Li F. (2019b). Citrinin monomer and dimer derivatives with antibacterial and cytotoxic activities isolated from the deep sea-derived fungus *Penicillium citrinum* NLG-S01-P1. *Mar. Drugs* 17:46. 10.3390/md17010046 30634700 PMC6357177

[B28] Wang W. Qin J. J. Voruganti S. Nijampatnam B. Velu S. E. Ruan K. H. (2018c). Discovery and characterization of dual inhibitors of MDM2 and NFAT1 for pancreatic cancer therapy. *Cancer Res.* 78 5656–5667. 10.1158/0008-5472.CAN-17-3939 30217928 PMC6435280

[B29] Wang W. Qin J. J. Voruganti S. Wang M. H. Sharma H. Patil S. (2014). Identification of a new class of MDM2 inhibitor that inhibits growth of orthotopic pancreatic tumors in mice. *Gastroenterol.* 147 893–902.e2. 10.1053/j.gastro.2014.07.001 25016295 PMC4170027

[B30] Wang W. Yang J. Liao Y. Y. Cheng G. Chen J. Cheng X. D. (2020a). Cytotoxic nitrogenated azaphilones from the deep-sea-derived fungus *Chaetomium globosum* MP4-S01-7. *J. Nat. Prod.* 83 1157–1166. 10.1021/acs.jnatprod.9b01165 32193933

[B31] Wang W. Yang J. Liao Y. Y. Cheng G. Chen J. Mo S. (2020b). Aspeterreurone A, a cytotoxic dihydrobenzofuran-phenyl acrylate hybrid from the deep-sea-derived fungus *Aspergillus terreus* CC-S06-18. *J. Nat. Prod.* 83 1998–2003. 10.1021/acs.jnatprod.0c00189 32489099

[B32] Wang W.-L. Lu Z.-Y. Tao H.-W. Zhu T.-J. Fang Y.-C. Gu Q.-Q. (2007). Isoechinulin-type alkaloids, variecolorins A-L, from halotolerant *Aspergillus variecolor*. *J. Nat. Prod.* 70 1558–1564. 10.1021/np070208z 17896816

[B33] Wei X. Feng C. Wang S.-Y. Zhang D.-M. Li X.-H. Zhang C.-X. (2020). New indole diketopiperazine alkaloids from soft coral-associated epiphytic fungus *Aspergillus* sp. EGF 15-0-3. *Chem. Biodivers.* 17:e2000106. 10.1002/cbdv.202000106 32212241

[B34] Wen H. Liu X. Zhang Q. Deng Y. Zang Y. Wang J. (2018). Three new indole diketopiperazine alkaloids from *Aspergillus ochraceus*. *Chem. Biodivers.* 15:e1700550. 10.1002/cbdv.201700550 29479805

[B35] Willoughby P. H. Jansma M. J. Hoye T. R. (2014). A guide to small-molecule structure assignment through computation of ((1)H and (1)(3)C) NMR chemical shifts. *Nat. Protoc.* 9 643–660. 10.1038/nprot.2014.042 24556787

[B36] Yan L.-H. Li P.-H. Li X.-M. Yang S.-Q. Liu K.-C. Wang B.-G. (2022). Chevalinulins A and B, proangiogenic alkaloids with a spiro[bicyclo[2.2.2]octane-diketopiperazine] skeleton from deep-sea cold-seep-derived fungus *Aspergillus chevalieri* CS-122. *Org. Lett.* 24 2684–2688. 10.1021/acs.orglett.2c00781 35389665

[B37] Yang J. Gong L. Guo M. Jiang Y. Ding Y. Wang Z. (2021). Bioactive indole diketopiperazine alkaloids from the marine endophytic fungus *Aspergillus* sp. YJ191021. *Mar. Drugs* 19:157. 10.3390/md19030157 33802820 PMC8002477

[B38] Zhang Q. Wang S.-Q. Tang H.-Y. Li X.-J. Zhang L. Xiao J. (2013). Potential allelopathic indole diketopiperazines produced by the plant endophytic aspergillus fumigatus using OSMAC method. *J. Agric. Food Chem.* 61 11447–11452. 10.1021/jf403200g 24188331

[B39] Zou X. Li Y. Zhang X. Li Q. Liu X. Huang Y. (2014). A new prenylated indole diketopiperazine alkaloid from *Eurotium cristatum*. *Molecules* 19 17839–17847. 10.3390/molecules191117839 25372398 PMC6271712

