# Supplementary material for: Indole Diketopiperazine Alkaloids Isolated From the Marine-Derived Fungus Aspergillus chevalieri MCCC M23426
Source: Front Microbiol. 2022 Jul 7;13:950857. doi: 10.3389/fmicb.2022.950857 (PMC9301495; doi:10.3389/fmicb.2022.950857)
Supplement: Supplementary file 1 [file Data_Sheet_1.docx]

Table of Content

[Figure S1. MS spectrum of 1 4](#_Toc106464914)

[Figure S2. ^13^C/DEPT spectrum of 1 in chloroform-*d* 4](#_Toc106464915)

[Figure S3. HSQC spectrum of 1 in chloroform-*d* 5](#_Toc106464916)

[Figure S4. UV spectrum of 1 5](#_Toc106464917)

[Figure S5. HMBC spectrum of 1 in chloroform-*d* 6](#_Toc106464918)

[Figure S6. ^13^C NMR spectrum of 1 in chloroform-*d* 6](#_Toc106464919)

[Figure S7. COSY spectrum of 1 in chloroform-*d* 7](#_Toc106464920)

[Figure S8. ^1^H NMR spectrum of 1 in chloroform-*d* 8](#_Toc106464921)

[Figure S9. Results of DP4+ analysis 8](#_Toc106464922)

[Figure S10. UV spectrum of 2 8](#_Toc106464923)

[Figure S11. MS spectrum of 2 9](#_Toc106464924)

[Figure S12. ^13^C NMR spectrum of 2 in chloroform-*d* 9](#_Toc106464925)

[Figure S13. ^13^C/DEPT spectrum of 2 in chloroform-*d* 10](#_Toc106464926)

[Figure S14. HSQC spectrum of 2 in chloroform-*d* 11](#_Toc106464927)

[Figure S15. HMBC spectrum of 2 in chloroform-*d* 11](#_Toc106464928)

[Figure S16. COSY spectrum of 2 in chloroform-*d* 12](#_Toc106464929)

[Figure S17. ^1^H NMR spectrum of 2 in chloroform-*d* 12](#_Toc106464930)

[Figure S18. ^1^H NMR spectrum of 3 in chloroform-*d* 13](#_Toc106464931)

[Figure S19. ^13^C NMR spectrum of 3 in chloroform-*d* 14](#_Toc106464932)

[Figure S20. ^13^C/DEPT spectrum of 3 in chloroform-*d* 14](#_Toc106464933)

[Figure S21. HSQC spectrum of 3 in chloroform-*d* 15](#_Toc106464934)

[Figure S22. HMBC spectrum of 3 in chloroform-*d* 15](#_Toc106464935)

[Figure S23. COSY spectrum of 3 in chloroform-*d* 16](#_Toc106464936)

[Table S1. Experimental and calculated ^13^C-NMR chemical shifts of (8*E*)-1 16](#_Toc106464937)

[Table S2. Experimental and calculated ^1^H-NMR chemical shifts of (8*E*)-1 17](#_Toc106464938)

[Table S3. Conformational analysis of the B3lyp/6-31G(d) optimized conformers of (8*E*)-1 in the gas phase (T=298.15 K) 17](#_Toc106464939)

[Table S4. Atomic coordinates (Å) of (8*E*)-1-1 obtained at the B3lyp/6-31G(d) level of theory in the gas phase. 17](#_Toc106464940)

[Table S5. Atomic coordinates (Å) of (8*E*)-1-2 obtained at the B3lyp/6-31G(d) level of theory in the gas phase. 18](#_Toc106464941)

[Table S6. Atomic coordinates (Å) of (8*E*)-1-3 obtained at the B3lyp/6-31G(d) level of theory in the gas phase. 19](#_Toc106464942)

[Table S7. Atomic coordinates (Å) of (8*E*)-1-4 obtained at the B3lyp/6-31G(d) level of theory in the gas phase. 20](#_Toc106464943)

[Table S8. Atomic coordinates (Å) of (8*E*)-1-5 obtained at the B3lyp/6-31G(d) level of theory in the gas phase. 20](#_Toc106464944)

[Table S9. Atomic coordinates (Å) of (8*E*)-1-6 obtained at the B3lyp/6-31G(d) level of theory in the gas phase. 21](#_Toc106464945)

[Table S10. Experimental and calculated ^13^C-NMR chemical shifts of (8*Z*)-1 22](#_Toc106464946)

[Table S11. Experimental and calculated ^1^H-NMR chemical shifts of (8*Z*)-1 22](#_Toc106464947)

[Table S12. Conformational analysis of the B3lyp/6-31G(d) optimized conformers of (8*Z*)-1 in the gas phase (T=298.15 K) 23](#_Toc106464948)

[Table S13. Atomic coordinates (Å) of (8*Z*)-1-1 obtained at the B3lyp/6-31G(d) level of theory in the gas phase. 23](#_Toc106464949)

[Table S14. Atomic coordinates (Å) of (8*Z*)-1-2 obtained at the B3lyp/6-31G(d) level of theory in the gas phase. 24](#_Toc106464950)

[Table S15. Atomic coordinates (Å) of (8*Z*)-1-3 obtained at the B3lyp/6-31G(d) level of theory in the gas phase. 24](#_Toc106464951)

[Table S16. Conformational analysis of the B3lyp/6-31G(d) optimized conformers of (9*R*)-2 in the gas phase (T=298.15 K) 25](#_Toc106464952)

[Table S17. Key transitions, oscillator strengths, and rotatory strengths in the ECD spectrum of conformer (9*R*)-2-1 at the B3lyp/6-31G(d) level of theory in MeOH with IEFPCM solvent model. 25](#_Toc106464953)

[Table S18. Key transitions, oscillator strengths, and rotatory strengths in the ECD spectrum of conformer (9*R*)-2-2 at the B3lyp/6-31G(d) level of theory in MeOH with IEFPCM solvent model. 27](#_Toc106464954)

[Table S19. Key transitions, oscillator strengths, and rotatory strengths in the ECD spectrum of conformer (9*R*)-2-3 at the B3lyp/6-31G(d) level of theory in MeOH with IEFPCM solvent model. 29](#_Toc106464955)

[Table S20. Key transitions, oscillator strengths, and rotatory strengths in the ECD spectrum of conformer (9*R*)-2-4 at the B3lyp/6-31G(d) level of theory in MeOH with IEFPCM solvent model. 30](#_Toc106464956)

[Table S21. Key transitions, oscillator strengths, and rotatory strengths in the ECD spectrum of conformer (9*R*)-2-5 at the B3lyp/6-31G(d) level of theory in MeOH with IEFPCM solvent model. 31](#_Toc106464957)

[Table S22. Key transitions, oscillator strengths, and rotatory strengths in the ECD spectrum of conformer (9*R*)-2-6 at the B3lyp/6-31G(d) level of theory in MeOH with IEFPCM solvent model. 33](#_Toc106464958)

[Table S23. Conformational analysis of the B3lyp/6-31G(d) optimized conformers of (12*S*28*S*31*R*)-3 in the gas phase (T=298.15 K) 34](#_Toc106464959)

[Table S24. Key transitions, oscillator strengths, and rotatory strengths in the ECD spectrum of conformer (12*S*28*S*31*R*)-3-1 at the B3lyp/6-31G(d) level of theory in MeOH with IEFPCM solvent model. 34](#_Toc106464960)

[Table S25. Key transitions, oscillator strengths, and rotatory strengths in the ECD spectrum of conformer (12*S*28*S*31*R*)-3-2 at the B3lyp/6-31G(d) level of theory in MeOH with IEFPCM solvent model. 36](#_Toc106464961)

[Table S26. Key transitions, oscillator strengths, and rotatory strengths in the ECD spectrum of conformer (12*S*28*S*31*R*)-3-3 at the B3lyp/6-31G(d) level of theory in MeOH with IEFPCM solvent model. 37](#_Toc106464962)

[Table S27. Key transitions, oscillator strengths, and rotatory strengths in the ECD spectrum of conformer (12*S*28*S*31*R*)-3-4 at the B3lyp/6-31G(d) level of theory in MeOH with IEFPCM solvent model. 39](#_Toc106464963)

[Fermentation, Extraction, and Isolation 40](#_Toc106464964)

[NMR Calculation 41](#_Toc106464965)


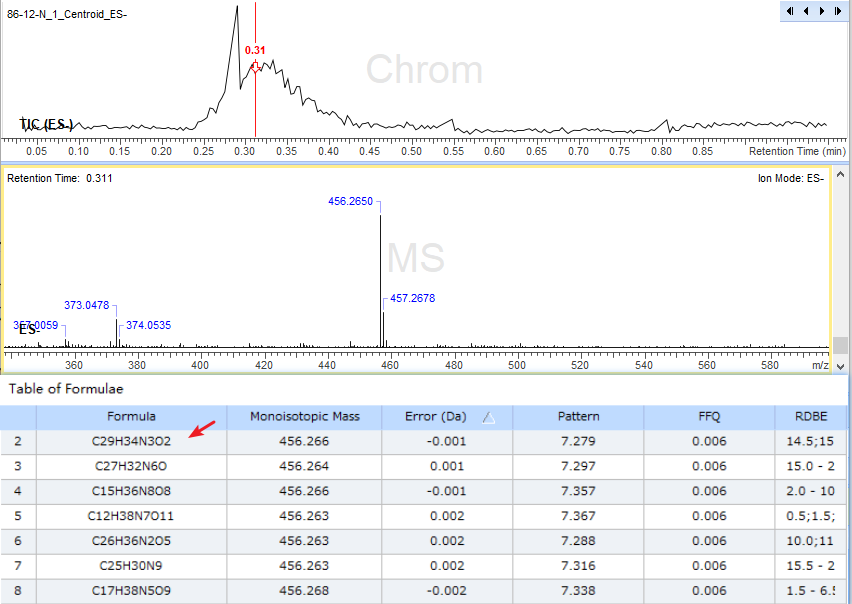


1. MS spectrum of **1**

1. ^13^C/DEPT spectrum of **1** in chloroform-*d*

1. HSQC spectrum of **1** in chloroform-*d*


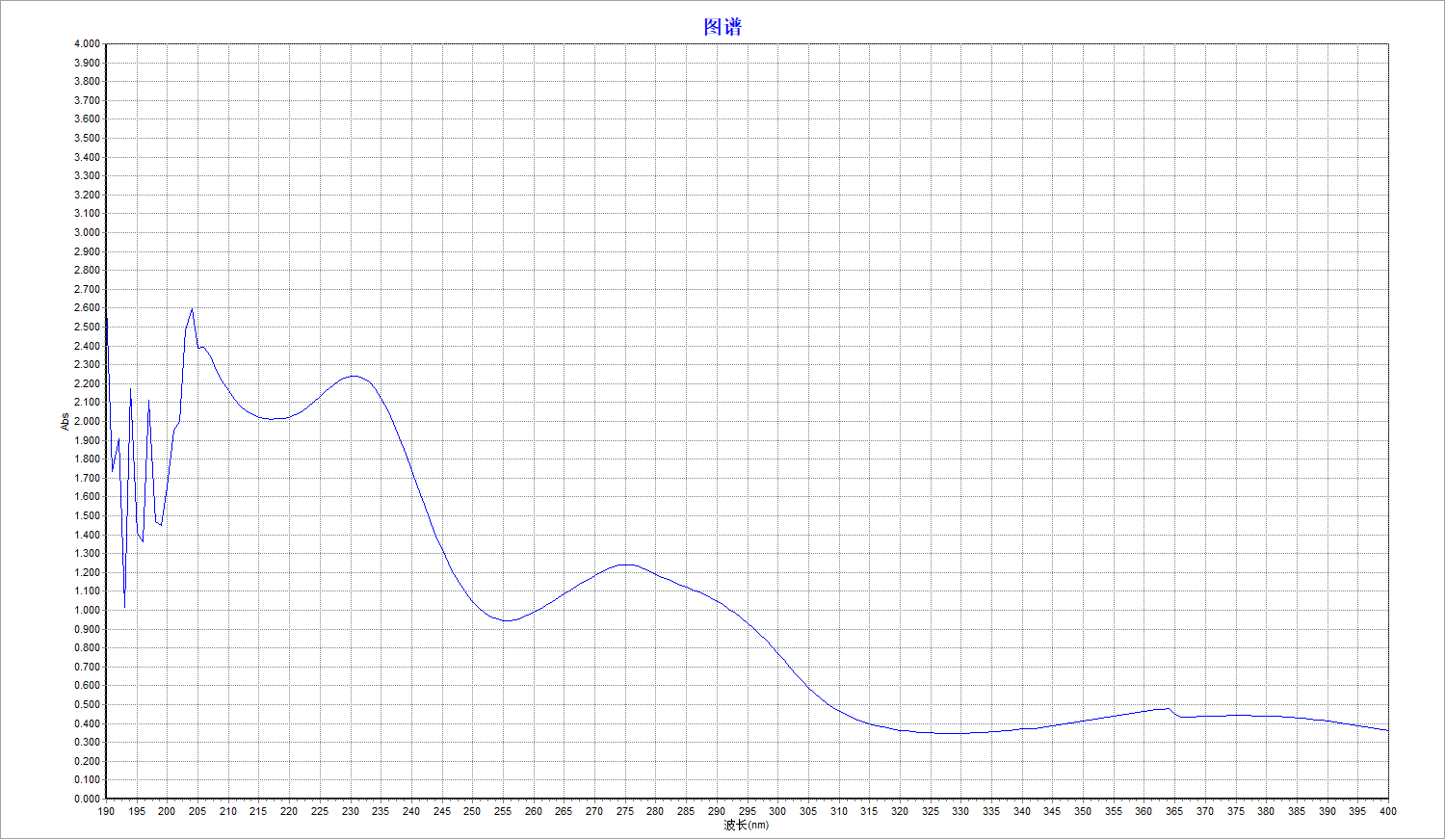


1. UV spectrum of **1**

1. HMBC spectrum of **1** in chloroform-*d*

1. ^13^C NMR spectrum of **1** in chloroform-*d*

1. COSY spectrum of **1** in chloroform-*d*

1. ^1^H NMR spectrum of **1** in chloroform-*d*


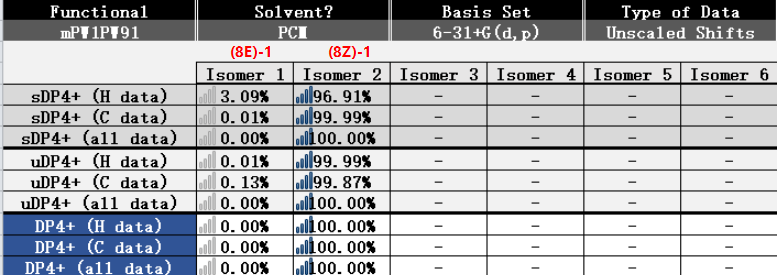


1. Results of DP4+ analysis


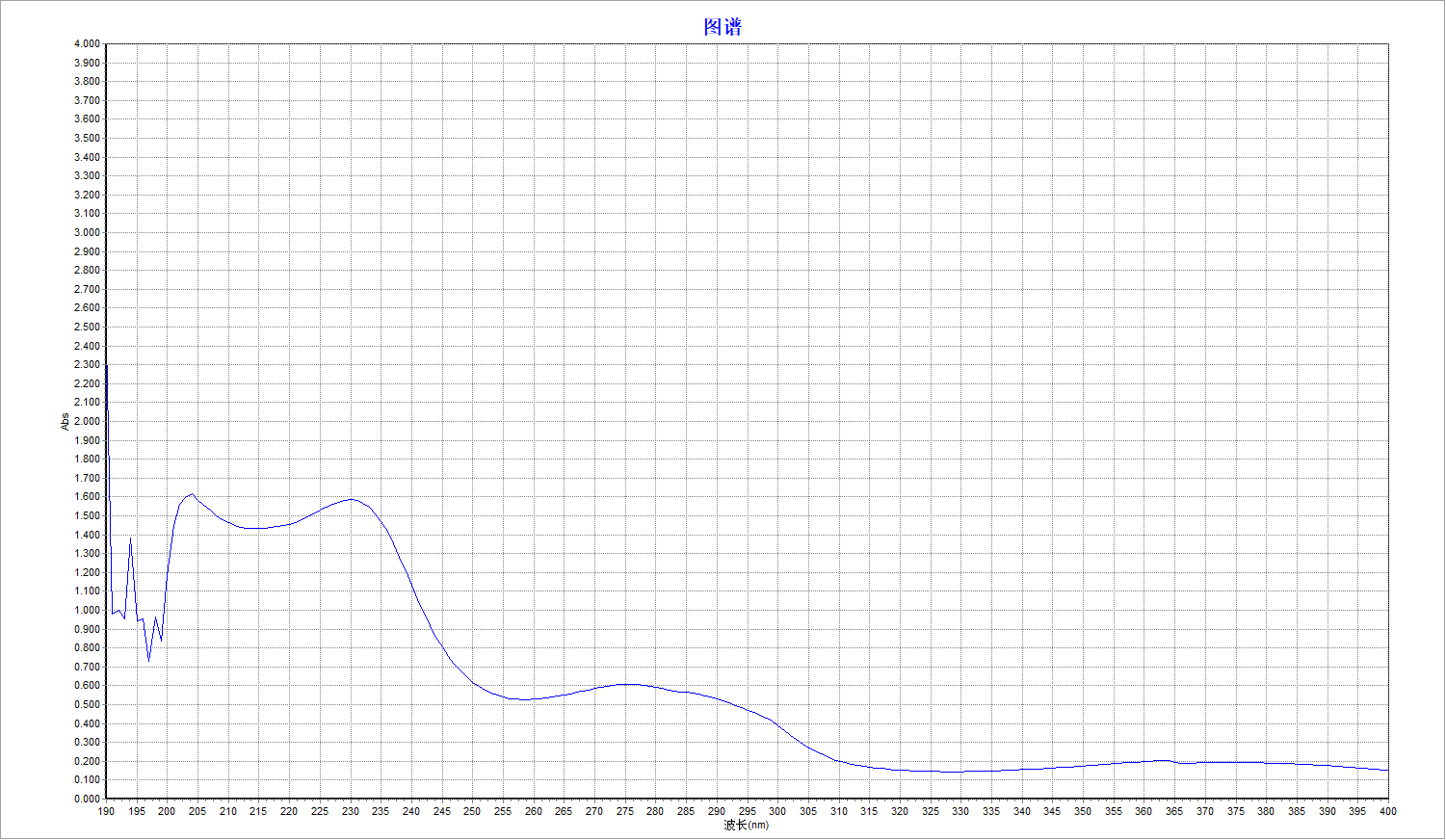


1. UV spectrum of **2**


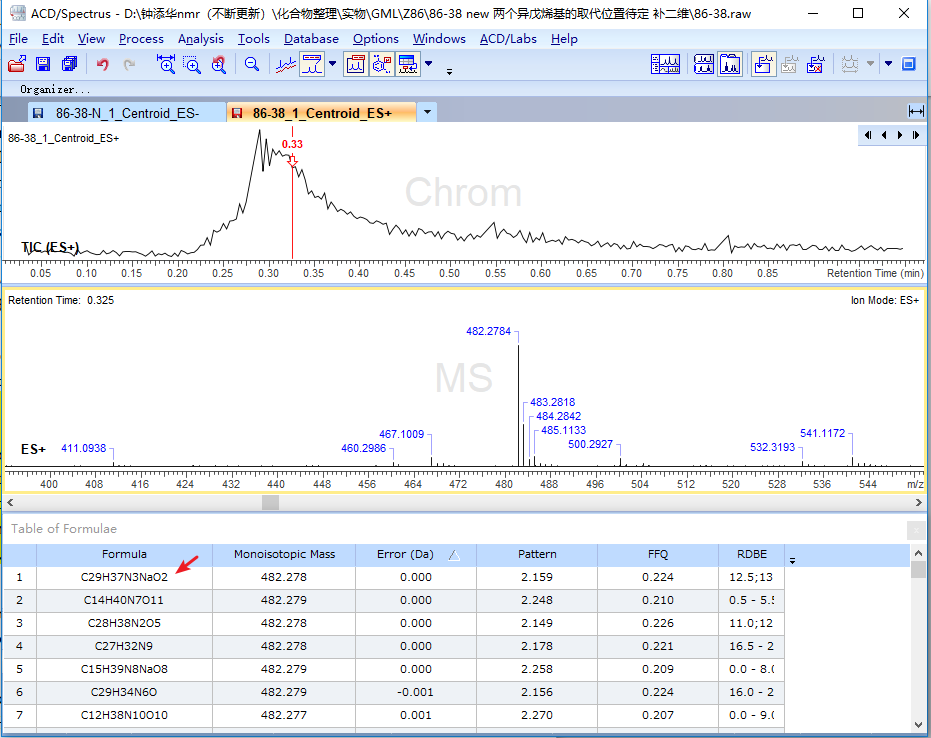


1. MS spectrum of **2**

1. ^13^C NMR spectrum of **2** in chloroform-*d*

1. ^13^C/DEPT spectrum of **2** in chloroform-*d*

1. HSQC spectrum of **2** in chloroform-*d*

1. HMBC spectrum of **2** in chloroform-*d*

1. COSY spectrum of **2** in chloroform-*d*

1. ^1^H NMR spectrum of **2** in chloroform-*d*

1. ^1^H NMR spectrum of **3** in chloroform-*d*

1. ^13^C NMR spectrum of **3** in chloroform-*d*

1. ^13^C/DEPT spectrum of **3** in chloroform-*d*

1. HSQC spectrum of **3** in chloroform-*d*

1. HMBC spectrum of **3** in chloroform-*d*

1. COSY spectrum of **3** in chloroform-*d*
2. Experimental and calculated ^13^C-NMR chemical shifts of (8*E*)-**1**

| No. | δexptl. | (8E)-1-δcalcd. |
| --- | --- | --- |
| 2 | 143.9 | 140.2 |
| 3 | 102.8 | 109.6 |
| 4 | 118.8 | 116.8 |
| 5 | 133.5 | 130.5 |
| 6 | 134.9 | 132.6 |
| 7 | 111.1 | 108.6 |
| 8 | 113.6 | 118.0 |
| 9 | 123.7 | 122.6 |
| 10 | 157.7 | 150.8 |
| 12 | 133.6 | 133.3 |
| 13 | 155.7 | 151.1 |
| 15 | 39.3 | 43.4 |
| 16 | 144.3 | 144.8 |
| 17 | 113.3 | 109.4 |
| 18 | 27.5 | 26.2 |
| 19 | 27.5 | 30.5 |
| 20 | 101.6 | 97.4 |
| 21 | 31.9 | 33.7 |
| 22 | 123.5 | 121.6 |
| 23 | 132.4 | 137.3 |
| 24 | 17.8 | 19.3 |
| 25 | 25.6 | 26.8 |
| 26 | 31.7 | 37.6 |
| 27 | 123.1 | 124.7 |
| 28 | 132.6 | 130.8 |
| 29 | 25.8 | 26.6 |
| 30 | 17.9 | 18.9 |
| 7a | 133.3 | 130.3 |
| 3a | 124.1 | 124.6 |

1. Experimental and calculated ^1^H-NMR chemical shifts of (8*E*)-**1**

| No. | δexptl. | (8E)-1-δcalcd. |
| --- | --- | --- |
| 4 | 7.07 | 7.15 |
| 7 | 7.17 | 7.27 |
| 8 | 7.28 | 6.74 |
| 16 | 6.05 | 6.27 |
| 17 | 5.21 | 5.19 |
| 17 | 5.17 | 4.93 |
| 18 | 1.51 | 1.46 |
| 19 | 1.51 | 1.31 |
| 20 | 5.6 | 5.61 |
| 20 | 4.94 | 4.85 |
| 21 | 3.39 | 3.39 |
| 22 | 5.27 | 5.68 |
| 24 | 1.69 | 1.51 |
| 25 | 1.72 | 1.71 |
| 26 | 3.4 | 3.33 |
| 27 | 5.31 | 5.21 |
| 29 | 1.78 | 1.62 |
| 30 | 1.71 | 1.68 |

1. Conformational analysis of the B3lyp/6-31G(d) optimized conformers of (8*E*)-**1** in the gas phase (T=298.15 K)

| Conformer | ^a^E (Hartree) | ^b^C (Hartree) | ^c^G (kcal/mol) | ΔG (kcal/mol) | Population |
| --- | --- | --- | --- | --- | --- |
| (8E)-1-1 | -1440.655523 | 0.506061 | -903693.787694 | 0.0 | 47.13% |
| (8E)-1-2 | -1440.654741 | 0.505844 | -903693.43283 | 0.354864 | 25.88% |
| (8E)-1-3 | -1440.652114 | 0.503496 | -903693.257588 | 0.530106 | 19.25% |
| (8E)-1-4 | -1440.650757 | 0.503918 | -903692.141234 | 1.64646 | 2.92% |
| (8E)-1-5 | -1440.651493 | 0.50474 | -903692.087388 | 1.700305 | 2.67% |
| (8E)-1-6 | -1440.651706 | 0.505157 | -903691.959422 | 1.828271 | 2.15% |

^a^Electronic energy obtained at M062X/6-311+G(2d,p) level of theory; ^b^Thermal correction to Gibbs free energy obtained at B3lyp/6-31G(d) level of theory; ^c^Gibbs free energy (E + C); The relative Gibbs free energy; The Boltzmann distribution of each conformer.

1. Atomic coordinates (Å) of (8*E*)-**1**-1 obtained at the B3lyp/6-31G(d) level of theory in the gas phase.

| N | -0.697918 | -3.349409 | 0.168883 | H | 0.309350 | 0.786981 | -0.973744 |
| --- | --- | --- | --- | --- | --- | --- | --- |
| C | -1.882366 | -2.641854 | 0.162720 | H | 2.121762 | -3.781944 | -0.108589 |
| C | -1.599991 | -1.313588 | -0.154476 | H | -3.481579 | -0.580491 | -0.901085 |
| C | 0.692196 | -0.206176 | -0.779180 | H | -0.974452 | 3.361639 | 1.769836 |
| C | 2.052175 | -0.449238 | -0.925789 | H | -4.240457 | 1.529675 | -1.105282 |
| C | 2.575190 | -1.749416 | -0.665900 | H | -5.176662 | -3.601948 | -0.412566 |
| C | 1.723881 | -2.786195 | -0.290408 | H | -4.607035 | -3.161158 | -2.730502 |
| C | -2.586173 | -0.273153 | -0.358270 | H | -2.853403 | -2.790443 | -2.257761 |
| C | -2.530887 | 1.040709 | -0.019287 | H | 2.732793 | 3.297378 | 1.589657 |
| C | -1.552219 | 1.625139 | 0.928602 | H | 1.420734 | 2.106616 | 1.484046 |
| N | -1.644675 | 2.993619 | 1.104974 | H | 3.066087 | 1.623501 | 1.099728 |
| C | -2.547095 | 3.882006 | 0.513001 | H | 4.221963 | -3.105539 | -0.798268 |
| C | -3.611126 | 3.276575 | -0.344363 | H | 4.499058 | -1.594899 | -1.645718 |
| N | -3.531300 | 1.915328 | -0.491980 | H | -2.379815 | -4.818245 | 1.860156 |
| C | -3.203538 | -3.293535 | 0.513078 | H | -2.562225 | -5.367172 | 0.166787 |
| C | -4.156716 | -3.316347 | -0.674996 | H | -3.976238 | -5.205291 | 1.208347 |
| C | -3.855208 | -3.078304 | -1.950689 | H | -4.044355 | -1.475542 | 1.416250 |
| C | -3.007256 | -4.759955 | 0.962593 | H | -4.823468 | -2.981794 | 1.945984 |
| C | -3.865072 | -2.520030 | 1.680818 | H | -3.216073 | -2.536036 | 2.562122 |
| C | -2.508277 | 5.209305 | 0.692115 | H | 3.839014 | 0.694064 | -0.659825 |
| C | 2.998096 | 0.667806 | -1.360968 | H | 3.438395 | 0.402635 | -2.332397 |
| C | 2.384472 | 2.041638 | -1.462447 | H | 2.128562 | 2.389754 | -2.462692 |
| C | 2.099876 | 2.840049 | -0.423623 | H | 2.058843 | 4.990013 | -0.192379 |
| C | 2.351458 | 2.446204 | 1.010395 | H | 1.298128 | 4.407628 | -1.688571 |
| C | 1.450502 | 4.185033 | -0.627772 | H | 0.470007 | 4.229747 | -0.134405 |
| C | 4.068803 | -2.019236 | -0.733681 | H | -3.257991 | 5.822121 | 0.209758 |
| C | 4.775554 | -1.495990 | 0.494567 | H | -1.753516 | 5.686710 | 1.308635 |
| C | 5.820482 | -0.658386 | 0.565699 | H | 7.427022 | -0.489524 | 2.005966 |
| C | 6.366301 | -0.221415 | 1.902845 | H | 6.309323 | 0.870786 | 2.011023 |
| C | 6.541420 | -0.075063 | -0.622885 | H | 5.817137 | -0.673668 | 2.734189 |
| C | 0.361574 | -2.517508 | -0.153928 | H | 7.604198 | -0.352318 | -0.601662 |
| C | -0.170924 | -1.229466 | -0.371437 | H | 6.130432 | -0.400428 | -1.580643 |
| O | -0.726110 | 0.976742 | 1.558187 | H | 6.504883 | 1.022630 | -0.596561 |
| O | -4.495148 | 3.933624 | -0.878075 | H | 4.340254 | -1.843578 | 1.431869 |
| H | -0.614143 | -4.319209 | 0.426254 | - | - | - | - |

1. Atomic coordinates (Å) of (8*E*)-**1**-2 obtained at the B3lyp/6-31G(d) level of theory in the gas phase.

| N | 0.821202 | -3.325601 | -0.214799 | H | -0.268350 | 0.784950 | -1.351543 |
| --- | --- | --- | --- | --- | --- | --- | --- |
| C | 2.003069 | -2.607968 | -0.300637 | H | -2.015530 | -3.769471 | -0.290143 |
| C | 1.689725 | -1.300995 | -0.653146 | H | 3.375328 | -0.315508 | -1.626532 |
| C | -0.639383 | -0.209208 | -1.130256 | H | 1.046056 | 2.953911 | 1.958767 |
| C | -2.008619 | -0.445748 | -1.148638 | H | 3.980948 | 1.861107 | -1.577055 |
| C | -2.507952 | -1.743496 | -0.838687 | H | 4.233075 | -3.779294 | 2.076008 |
| C | -1.630360 | -2.779714 | -0.524290 | H | 2.940067 | -2.345244 | 3.542397 |
| C | 2.588464 | -0.179614 | -0.884013 | H | 1.955926 | -1.699257 | 2.111912 |
| C | 2.503954 | 1.060779 | -0.345086 | H | -2.659003 | 2.788081 | 1.825531 |
| C | 1.633363 | 1.412855 | 0.806920 | H | -2.964669 | 1.210159 | 1.069297 |
| N | 1.644015 | 2.745559 | 1.167502 | H | -1.319674 | 1.681954 | 1.469609 |
| C | 2.378904 | 3.790094 | 0.598302 | H | -4.518352 | -1.577950 | -1.629426 |
| C | 3.342390 | 3.416148 | -0.480921 | H | -4.163115 | -3.096600 | -0.827156 |
| N | 3.344687 | 2.086777 | -0.820665 | H | 2.544920 | -5.331262 | 0.261002 |
| C | 3.295988 | -3.254096 | 0.148219 | H | 4.282256 | -5.191598 | 0.030177 |
| C | 3.441877 | -3.149859 | 1.664082 | H | 3.188498 | -4.869517 | -1.330602 |
| C | 2.736412 | -2.362549 | 2.474880 | H | 5.436629 | -3.106253 | -0.187598 |
| C | 3.320247 | -4.750000 | -0.250257 | H | 4.466991 | -2.614772 | -1.589790 |
| C | 4.526765 | -2.579935 | -0.495702 | H | 4.621122 | -1.540702 | -0.174945 |
| C | 2.266348 | 5.069546 | 0.979201 | H | -3.368010 | 0.516560 | -2.514166 |
| C | -2.979012 | 0.679429 | -1.499031 | H | -3.846701 | 0.602509 | -0.837548 |
| C | -2.413306 | 2.076723 | -1.405237 | H | -2.228021 | 2.597749 | -2.343770 |
| C | -2.107105 | 2.703486 | -0.260290 | H | -1.423762 | 4.492742 | -1.274890 |
| C | -2.279644 | 2.061915 | 1.094549 | H | -2.150230 | 4.785680 | 0.319262 |
| C | -1.524248 | 4.093176 | -0.260807 | H | -0.528833 | 4.107304 | 0.202447 |
| C | -4.002667 | -2.011188 | -0.767342 | H | 2.884901 | 5.813308 | 0.494921 |
| C | -4.581786 | -1.499347 | 0.530797 | H | 1.579658 | 5.381522 | 1.759350 |
| C | -5.614854 | -0.664437 | 0.717295 | H | -5.380451 | -0.693089 | 2.872682 |
| C | -6.013419 | -0.233773 | 2.107399 | H | -5.939034 | 0.857481 | 2.214756 |
| C | -6.460445 | -0.077614 | -0.384230 | H | -7.058315 | -0.498005 | 2.321604 |
| C | -0.261161 | -2.513103 | -0.497045 | H | -6.154940 | -0.398934 | -1.382111 |
| C | 0.249972 | -1.232142 | -0.788777 | H | -7.514084 | -0.357779 | -0.249605 |
| O | 0.954613 | 0.611571 | 1.437344 | H | -6.423829 | 1.020088 | -0.357146 |
| O | 4.081666 | 4.223325 | -1.027233 | H | -4.049460 | -1.850702 | 1.415107 |
| H | 0.757326 | -4.261663 | 0.150536 | - | - | - | - |

1. Atomic coordinates (Å) of (8*E*)-**1**-3 obtained at the B3lyp/6-31G(d) level of theory in the gas phase.

| N | -0.504102 | 2.571107 | 0.535853 | H | 0.685014 | -1.422540 | -0.883601 |
| --- | --- | --- | --- | --- | --- | --- | --- |
| C | -1.665984 | 1.939022 | 0.120086 | H | 2.301461 | 2.892937 | 1.044119 |
| C | -1.323855 | 0.665706 | -0.323371 | H | -1.745767 | -0.758893 | -1.876003 |
| C | 1.031389 | -0.473859 | -0.480450 | H | -5.395366 | -1.289264 | 2.039021 |
| C | 2.389640 | -0.287963 | -0.235503 | H | -3.526902 | -2.085943 | -2.223450 |
| C | 2.852043 | 0.939477 | 0.319848 | H | -3.733454 | 1.842019 | -1.708712 |
| C | 1.947514 | 1.957879 | 0.616629 | H | -5.997444 | 1.357082 | -1.015766 |
| C | -2.128434 | -0.385198 | -0.921860 | H | -5.650476 | 1.964456 | 0.692791 |
| C | -3.256384 | -0.984032 | -0.477865 | H | 7.151845 | -2.509350 | 0.079797 |
| C | -3.829981 | -0.794572 | 0.879128 | H | 6.188157 | -3.786591 | -0.652814 |
| N | -5.033391 | -1.431871 | 1.103317 | H | 5.921939 | -2.100774 | -1.127405 |
| C | -5.766949 | -2.230447 | 0.221773 | H | 4.433645 | 2.091632 | 1.170954 |
| C | -5.137809 | -2.510006 | -1.104477 | H | 4.723552 | 0.360155 | 1.200636 |
| N | -3.923691 | -1.904894 | -1.308154 | H | -4.298473 | 3.594754 | 1.640158 |
| C | -2.964172 | 2.730328 | 0.148894 | H | -2.612065 | 3.484781 | 2.162131 |
| C | -4.036528 | 2.055939 | -0.684447 | H | -3.602092 | 2.017946 | 2.109487 |
| C | -5.287819 | 1.787955 | -0.314994 | H | -3.667850 | 4.667108 | -0.563411 |
| C | -3.402040 | 2.965893 | 1.608566 | H | -1.999969 | 4.718973 | 0.017419 |
| C | -2.726877 | 4.109918 | -0.532109 | H | -2.360741 | 3.988775 | -1.556848 |
| C | -6.964010 | -2.755550 | 0.514922 | H | 2.815679 | -2.180117 | -1.118996 |
| C | 3.355380 | -1.418220 | -0.538488 | H | 4.161430 | -1.055718 | -1.184950 |
| C | 3.932758 | -2.058038 | 0.703887 | H | 3.268016 | -2.059255 | 1.568015 |
| C | 5.135977 | -2.635339 | 0.834380 | H | 5.787813 | -4.330752 | 2.009194 |
| C | 6.144683 | -2.753270 | -0.280114 | H | 6.472582 | -2.793764 | 2.525112 |
| C | 5.557070 | -3.263936 | 2.139015 | H | 4.779239 | -3.175387 | 2.903694 |
| C | 4.327644 | 1.170900 | 0.579564 | H | -7.454483 | -3.377089 | -0.222300 |
| C | 5.142801 | 1.292883 | -0.689422 | H | -7.453392 | -2.582325 | 1.467932 |
| C | 6.445852 | 1.016050 | -0.840455 | H | 6.444934 | 1.589131 | -2.931549 |
| C | 7.132682 | 1.211172 | -2.168810 | H | 7.969836 | 1.918216 | -2.083235 |
| C | 7.341457 | 0.536132 | 0.272978 | H | 7.560761 | 0.266147 | -2.531852 |
| C | 0.593453 | 1.753327 | 0.353205 | H | 8.019435 | 1.339408 | 0.594884 |
| C | 0.114161 | 0.546620 | -0.199451 | H | 6.785418 | 0.196767 | 1.149901 |
| O | -3.294077 | -0.159479 | 1.777038 | H | 7.977330 | -0.290725 | -0.066738 |
| O | -5.652539 | -3.226668 | -1.952252 | H | 4.595794 | 1.669218 | -1.553994 |
| H | -0.466151 | 3.515136 | 0.884624 | - | - | - | - |

1. Atomic coordinates (Å) of (8*E*)-**1**-4 obtained at the B3lyp/6-31G(d) level of theory in the gas phase.

| N | -0.607549 | 2.749444 | -0.108024 | H | 0.549805 | -1.435048 | -0.866481 |
| --- | --- | --- | --- | --- | --- | --- | --- |
| C | -1.763996 | 2.066843 | -0.456888 | H | 2.183261 | 3.103158 | 0.439612 |
| C | -1.430217 | 0.746118 | -0.696285 | H | -2.296443 | -0.601736 | -2.187415 |
| C | 0.901037 | -0.443387 | -0.591407 | H | -4.328180 | -1.515424 | 2.719759 |
| C | 2.251431 | -0.239948 | -0.325357 | H | -3.920666 | -2.166264 | -1.944779 |
| C | 2.719254 | 1.054495 | 0.046275 | H | -3.649569 | 2.199345 | 1.496849 |
| C | 1.825860 | 2.118254 | 0.148228 | H | -5.869630 | 1.316566 | 1.101802 |
| C | -2.320820 | -0.336325 | -1.126320 | H | -5.718599 | 1.627109 | -0.710429 |
| C | -3.167756 | -1.050635 | -0.359259 | H | 5.663137 | -2.340224 | 0.305059 |
| C | -3.271525 | -0.901493 | 1.123497 | H | 6.221151 | -3.507559 | -0.905604 |
| N | -4.280619 | -1.635463 | 1.714475 | H | 7.167049 | -2.058517 | -0.586296 |
| C | -5.184767 | -2.511785 | 1.107568 | H | 4.800581 | 0.947461 | -0.473262 |
| C | -5.018215 | -2.733459 | -0.360924 | H | 4.345285 | 2.379613 | 0.434202 |
| N | -4.001053 | -2.019572 | -0.944612 | H | -2.611835 | 4.448579 | 0.774627 |
| C | -3.110467 | 2.769163 | -0.558546 | H | -3.948457 | 4.758884 | -0.335421 |
| C | -4.051168 | 2.186274 | 0.485543 | H | -2.287556 | 4.767970 | -0.950805 |
| C | -5.270951 | 1.692180 | 0.276744 | H | -4.605134 | 3.147047 | -2.105408 |
| C | -2.970755 | 4.277167 | -0.247011 | H | -2.940323 | 3.032519 | -2.709605 |
| C | -3.653835 | 2.616075 | -1.991864 | H | -3.814797 | 1.564264 | -2.243016 |
| C | -6.155643 | -3.154332 | 1.769921 | H | 3.801755 | -1.515828 | 0.455201 |
| C | 3.214488 | -1.404882 | -0.462084 | H | 2.621721 | -2.325804 | -0.559219 |
| C | 4.131049 | -1.278298 | -1.657298 | H | 3.681566 | -0.794842 | -2.525197 |
| C | 5.395441 | -1.711701 | -1.764804 | H | 6.503604 | -2.500204 | -3.447441 |
| C | 6.140784 | -2.436198 | -0.672438 | H | 5.573376 | -1.033660 | -3.817027 |
| C | 6.169670 | -1.532620 | -3.046838 | H | 7.077910 | -0.937024 | -2.877742 |
| C | 4.182327 | 1.294735 | 0.361879 | H | -6.803233 | -3.825967 | 1.222247 |
| C | 4.631681 | 0.633328 | 1.647445 | H | -6.310186 | -3.026227 | 2.836412 |
| C | 5.881797 | 0.276766 | 1.974840 | H | 6.639495 | -1.348813 | 3.180011 |
| C | 6.186603 | -0.355664 | 3.309491 | H | 6.912112 | 0.244152 | 3.877003 |
| C | 7.081270 | 0.499679 | 1.089814 | H | 5.286605 | -0.469094 | 3.921726 |
| C | 0.475955 | 1.895165 | -0.125942 | H | 7.721662 | 1.292134 | 1.502802 |
| C | -0.008092 | 0.619733 | -0.500195 | H | 7.700439 | -0.404595 | 1.036282 |
| O | -2.535522 | -0.203684 | 1.802386 | H | 6.811408 | 0.786195 | 0.070791 |
| O | -5.723185 | -3.495088 | -1.007498 | H | 3.841041 | 0.459580 | 2.377344 |
| H | -0.566800 | 3.730083 | 0.114736 | - | - | - | - |

1. Atomic coordinates (Å) of (8*E*)-**1**-5 obtained at the B3lyp/6-31G(d) level of theory in the gas phase.

| N | -0.729377 | 2.658553 | -0.227402 | H | 0.495357 | -1.529251 | -0.815597 |
| --- | --- | --- | --- | --- | --- | --- | --- |
| C | -1.878983 | 1.949470 | -0.540757 | H | 2.048814 | 3.072643 | 0.352289 |
| C | -1.530256 | 0.619206 | -0.717617 | H | -1.990926 | -1.081629 | -1.967465 |
| C | 0.827663 | -0.521852 | -0.576035 | H | -4.819485 | -1.402164 | 2.577253 |
| C | 2.174553 | -0.290543 | -0.308100 | H | -3.606399 | -2.637215 | -1.818373 |
| C | 2.619479 | 1.020039 | 0.028264 | H | -3.575770 | 2.788179 | 1.422191 |
| C | 1.709357 | 2.074032 | 0.088319 | H | -3.615245 | 5.197222 | 1.793009 |
| C | -2.311837 | -0.563620 | -1.058746 | H | -3.262630 | 5.515104 | 0.009748 |
| C | -3.277163 | -1.195267 | -0.352778 | H | 7.107653 | -2.076857 | -0.479346 |
| C | -3.727348 | -0.789707 | 1.005676 | H | 5.596935 | -2.352908 | 0.402563 |
| N | -4.553610 | -1.692430 | 1.643162 | H | 6.177517 | -3.540638 | -0.777031 |
| C | -5.064062 | -2.898821 | 1.153024 | H | 4.706105 | 0.929767 | -0.470868 |
| C | -4.697726 | -3.256365 | -0.250640 | H | 4.222082 | 2.380046 | 0.391905 |
| N | -3.867369 | -2.361847 | -0.878253 | H | -3.805476 | 4.378214 | -2.045281 |
| C | -3.148044 | 2.766728 | -0.728591 | H | -2.734933 | 3.192911 | -2.819618 |
| C | -3.409044 | 3.473862 | 0.593348 | H | -2.060643 | 4.408762 | -1.730458 |
| C | -3.423115 | 4.790917 | 0.803684 | H | -5.222689 | 2.580442 | -1.286562 |
| C | -2.919471 | 3.752160 | -1.897201 | H | -4.198127 | 1.297546 | -1.961111 |
| C | -4.381304 | 1.912703 | -1.075205 | H | -4.662004 | 1.264101 | -0.247043 |
| C | -5.851178 | -3.715610 | 1.865275 | H | 3.736675 | -1.519010 | 0.521775 |
| C | 3.154471 | -1.445267 | -0.402459 | H | 2.575569 | -2.377286 | -0.475025 |
| C | 4.078142 | -1.341606 | -1.594633 | H | 3.632268 | -0.881600 | -2.476951 |
| C | 5.345341 | -1.771052 | -1.682741 | H | 7.033127 | -1.016709 | -2.801213 |
| C | 6.085638 | -2.465316 | -0.567709 | H | 6.468048 | -2.594710 | -3.338299 |
| C | 6.128117 | -1.619130 | -2.963007 | H | 5.535798 | -1.140370 | -3.748879 |
| C | 4.076014 | 1.291265 | 0.349172 | H | -6.212701 | -4.623986 | 1.402042 |
| C | 4.524195 | 0.673412 | 1.656487 | H | -6.137852 | -3.495516 | 2.888508 |
| C | 5.774756 | 0.333408 | 1.999573 | H | 5.175625 | -0.349078 | 3.968594 |
| C | 6.077152 | -0.253003 | 3.355628 | H | 6.533953 | -1.248254 | 3.260890 |
| C | 6.976880 | 0.532496 | 1.112531 | H | 6.798613 | 0.368175 | 3.904968 |
| C | 0.367166 | 1.823527 | -0.196535 | H | 6.710267 | 0.778926 | 0.082253 |
| C | -0.095392 | 0.530787 | -0.529137 | H | 7.609131 | 1.344679 | 1.498790 |
| O | -3.401003 | 0.248524 | 1.567674 | H | 7.603091 | -0.368295 | 1.095721 |
| O | -5.101251 | -4.265274 | -0.811790 | H | 3.732087 | 0.520041 | 2.389423 |
| H | -0.747312 | 3.621668 | 0.073042 | - | - | - | - |

1. Atomic coordinates (Å) of (8*E*)-**1**-6 obtained at the B3lyp/6-31G(d) level of theory in the gas phase.

| N | 0.456990 | 3.161528 | -0.672720 | H | -0.310344 | -0.929228 | 0.779430 |
| --- | --- | --- | --- | --- | --- | --- | --- |
| C | 1.680361 | 2.635449 | -0.307451 | H | -2.372513 | 3.215423 | -1.080124 |
| C | 1.487746 | 1.328879 | 0.128960 | H | 3.196713 | 0.959420 | 1.399885 |
| C | -0.749063 | -0.029998 | 0.360861 | H | 1.869645 | -3.552080 | -1.208882 |
| C | -2.125169 | 0.029936 | 0.163995 | H | 4.180703 | -0.969700 | 2.001342 |
| C | -2.716334 | 1.208884 | -0.372731 | H | 3.889488 | 1.948996 | -1.682668 |
| C | -1.919717 | 2.307361 | -0.688735 | H | 6.075390 | 1.835025 | -0.646562 |
| C | 2.512746 | 0.477207 | 0.702040 | H | 5.578662 | 3.060976 | 0.640571 |
| C | 2.711890 | -0.852698 | 0.527561 | H | -0.545944 | -2.648914 | -1.581374 |
| C | 2.070328 | -1.664060 | -0.534905 | H | -1.064266 | -3.118156 | 0.033546 |
| N | 2.323713 | -3.021495 | -0.474594 | H | -1.087083 | -4.313196 | -1.274241 |
| C | 3.161692 | -3.702473 | 0.413617 | H | -4.417841 | 2.180850 | -1.218593 |
| C | 3.934316 | -2.862716 | 1.379730 | H | -4.617316 | 0.448654 | -1.058858 |
| N | 3.670333 | -1.518366 | 1.318605 | H | 3.772684 | 5.102909 | -1.546255 |
| C | 2.952384 | 3.459314 | -0.384200 | H | 2.051312 | 5.292698 | -1.207439 |
| C | 4.102567 | 2.558536 | -0.805274 | H | 2.593206 | 4.133562 | -2.447368 |
| C | 5.307609 | 2.487680 | -0.241291 | H | 3.342337 | 3.393252 | 1.773103 |
| C | 2.826493 | 4.560573 | -1.466415 | H | 4.077466 | 4.783775 | 0.947934 |
| C | 3.195485 | 4.135046 | 0.982287 | H | 2.331260 | 4.749312 | 1.253492 |
| C | 3.313547 | -5.033551 | 0.419402 | H | -2.378585 | -1.839548 | 1.162384 |
| C | -2.968114 | -1.193959 | 0.501550 | H | -3.849910 | -0.887193 | 1.072700 |
| C | -3.410313 | -1.971916 | -0.719879 | H | -4.408579 | -1.750311 | -1.094103 |
| C | -2.682211 | -2.867426 | -1.400726 | H | -2.577657 | -3.321059 | -3.508276 |
| C | -1.273465 | -3.259017 | -1.030844 | H | -3.258238 | -4.618762 | -2.532006 |
| C | -3.224561 | -3.525833 | -2.643556 | H | -4.233145 | -3.175396 | -2.884611 |
| C | -4.220611 | 1.330311 | -0.550856 | H | 3.999216 | -5.476075 | 1.129574 |
| C | -4.927596 | 1.567194 | 0.762312 | H | 2.774599 | -5.679310 | -0.266346 |
| C | -5.975700 | 0.907111 | 1.275489 | H | -5.989255 | 2.115813 | 3.076038 |
| C | -6.538952 | 1.284990 | 2.623372 | H | -7.595748 | 1.576085 | 2.544172 |
| C | -6.686732 | -0.239373 | 0.602505 | H | -6.502565 | 0.433692 | 3.317548 |
| C | -0.543209 | 2.222190 | -0.482372 | H | -6.283228 | -0.479819 | -0.382986 |
| C | 0.068169 | 1.056508 | 0.027123 | H | -6.630769 | -1.146511 | 1.219811 |
| O | 1.375981 | -1.201439 | -1.431077 | H | -7.754414 | -0.011532 | 0.480285 |
| O | 4.746338 | -3.326547 | 2.169343 | H | -4.501524 | 2.378573 | 1.353277 |
| H | 0.328913 | 4.055114 | -1.118972 | - | - | - | - |

1. Experimental and calculated ^13^C-NMR chemical shifts of (8*Z*)-**1**

| No. | δexptl. | (8*Z*)-**1**-δcalcd. |
| --- | --- | --- |
| 2 | 143.9 | 142.2 |
| 3 | 102.8 | 105.6 |
| 4 | 118.8 | 116.7 |
| 5 | 133.5 | 135.0 |
| 6 | 134.9 | 132.9 |
| 7 | 111.1 | 109.3 |
| 8 | 113.6 | 114.0 |
| 9 | 123.7 | 121.7 |
| 10 | 157.7 | 152.5 |
| 12 | 133.6 | 134.3 |
| 13 | 155.7 | 150.8 |
| 15 | 39.3 | 43.6 |
| 16 | 144.3 | 142.7 |
| 17 | 113.3 | 111.2 |
| 18 | 27.5 | 31.3 |
| 19 | 27.5 | 27.4 |
| 20 | 101.6 | 98.8 |
| 21 | 31.9 | 32.9 |
| 22 | 123.5 | 119.4 |
| 23 | 132.4 | 140.0 |
| 24 | 17.8 | 18.1 |
| 25 | 25.6 | 26.1 |
| 26 | 31.7 | 37.1 |
| 27 | 123.1 | 124.3 |
| 28 | 132.6 | 130.9 |
| 29 | 25.8 | 27.5 |
| 30 | 17.9 | 19.1 |
| 7a | 133.3 | 130.9 |
| 3a | 124.1 | 121.5 |

1. Experimental and calculated ^1^H-NMR chemical shifts of (8*Z*)-**1**

| No. | δexptl. | (8*Z*)-**1**-δcalcd. |
| --- | --- | --- |
| 4 | 7.07 | 7.46 |
| 7 | 7.17 | 7.36 |
| 8 | 7.28 | 7.62 |
| 16 | 6.05 | 6.12 |
| 17 | 5.21 | 5.33 |
| 17 | 5.17 | 5.12 |
| 18 | 1.51 | 1.27 |
| 19 | 1.51 | 1.4 |
| 20 | 5.6 | 5.63 |
| 20 | 4.94 | 4.97 |
| 21 | 3.39 | 3.45 |
| 22 | 5.27 | 5.88 |
| 24 | 1.69 | 1.63 |
| 25 | 1.72 | 1.66 |
| 26 | 3.4 | 3.33 |
| 27 | 5.31 | 5.24 |
| 29 | 1.78 | 1.61 |
| 30 | 1.71 | 1.7 |

1. Conformational analysis of the B3lyp/6-31G(d) optimized conformers of (8*Z*)-**1** in the gas phase (T=298.15 K)

| Conformer | ^a^E (Hartree) | ^b^C (Hartree) | ^c^G (kcal/mol) | ΔG (kcal/mol) | Population |
| --- | --- | --- | --- | --- | --- |
| (8*Z*)-**1**-1 | -1440.66905 | 0.507722 | -903701.233082 | 0.0 | 90.83% |
| (8*Z*)-**1**-2 | -1440.665721 | 0.50682 | -903699.710196 | 1.522886 | 6.94% |
| (8*Z*)-**1**-3 | -1440.660977 | 0.503145 | -903699.039511 | 2.193571 | 2.23% |

^a^Electronic energy obtained at M062X/6-311+G(2d,p) level of theory; ^b^Thermal correction to Gibbs free energy obtained at B3lyp/6-31G(d) level of theory; ^c^Gibbs free energy (E + C); The relative Gibbs free energy; The Boltzmann distribution of each conformer.

1. Atomic coordinates (Å) of (8*Z*)-**1**-1 obtained at the B3lyp/6-31G(d) level of theory in the gas phase.

| N | 1.340078 | -3.136955 | 0.350336 | H | -2.010112 | 4.037563 | -1.916590 |
| --- | --- | --- | --- | --- | --- | --- | --- |
| C | 2.399417 | -2.259829 | 0.241915 | H | -3.276763 | 4.516971 | -0.767347 |
| C | 1.906946 | -1.025197 | -0.179250 | H | -7.123202 | -1.389019 | 2.087785 |
| C | -0.551125 | -0.361805 | -0.855574 | H | -5.497482 | -1.258795 | 2.793280 |
| C | -1.853503 | -0.840220 | -0.948466 | H | -6.212993 | 0.109681 | 1.919436 |
| C | -2.155260 | -2.172993 | -0.544739 | H | -2.435990 | 2.302789 | 1.430511 |
| C | -1.143342 | -3.012942 | -0.086030 | H | -4.083161 | 2.755209 | 0.992401 |
| C | 2.700702 | 0.163812 | -0.418806 | H | -3.558921 | 1.065059 | 0.833134 |
| C | 2.363414 | 1.436179 | -0.103535 | H | -6.457809 | -0.059583 | -0.684279 |
| C | 3.279934 | 2.537877 | -0.485028 | H | -5.888366 | -1.514998 | -1.516461 |
| N | 2.859931 | 3.803567 | -0.129175 | H | -7.341679 | -1.572004 | -0.508069 |
| C | 1.692806 | 4.152564 | 0.556703 | H | 3.508289 | -4.834589 | 0.381931 |
| C | 0.806510 | 3.029494 | 0.995159 | H | 4.893404 | -4.387669 | 1.377179 |
| N | 1.207998 | 1.778577 | 0.616104 | H | 3.267056 | -4.226492 | 2.048081 |
| C | 3.813358 | -2.668341 | 0.598695 | H | 4.366525 | -0.689762 | 1.376639 |
| C | 4.743191 | -2.603604 | -0.606098 | H | 5.376912 | -2.018577 | 1.978635 |
| C | 4.396930 | -2.469731 | -1.884849 | H | 3.727980 | -1.798588 | 2.602593 |
| C | 4.354725 | -1.730648 | 1.707086 | H | -3.560840 | -3.783505 | -0.501578 |
| C | 3.861372 | -4.118746 | 1.132467 | H | -4.080033 | -2.434103 | -1.495908 |
| C | 1.349408 | 5.416384 | 0.840997 | H | -3.846156 | -0.070065 | -0.885245 |
| C | -2.960282 | 0.044017 | -1.513344 | H | -3.248682 | -0.348101 | -2.499987 |
| C | -2.625403 | 1.507700 | -1.647232 | H | 0.435818 | 5.598077 | 1.390730 |
| C | -2.759183 | 2.423876 | -0.677157 | H | 1.961365 | 6.261382 | 0.542497 |
| C | -3.242435 | 2.104410 | 0.714490 | H | 5.146824 | -2.465722 | -2.670434 |
| C | -2.399471 | 3.869417 | -0.907099 | H | 3.362051 | -2.344562 | -2.190258 |
| C | -3.585403 | -2.686197 | -0.553451 | H | -1.370685 | -4.036632 | 0.202186 |
| C | -4.359506 | -2.152534 | 0.629336 | H | 3.674280 | 0.054207 | -0.885529 |
| C | -5.520560 | -1.480973 | 0.636206 | H | -0.340458 | 0.651239 | -1.177865 |
| C | -6.113577 | -0.984581 | 1.931652 | H | -3.869835 | -2.326229 | 1.587948 |
| C | -6.332172 | -1.146772 | -0.589300 | H | -2.230792 | 1.831838 | -2.610033 |
| C | 0.157827 | -2.516821 | -0.009217 | H | 5.796899 | -2.717683 | -0.347895 |
| C | 0.475297 | -1.183934 | -0.359882 | H | 1.420851 | -4.092572 | 0.657886 |
| O | 4.338818 | 2.369546 | -1.077298 | H | 3.495175 | 4.541703 | -0.409358 |
| O | -0.213732 | 3.215708 | 1.650694 | H | 0.587575 | 1.028365 | 0.901872 |
| H | -1.647462 | 4.197629 | -0.179790 | - | - | - | - |

1. Atomic coordinates (Å) of (8*Z*)-**1**-2 obtained at the B3lyp/6-31G(d) level of theory in the gas phase.

| N | -1.173961 | -2.929021 | -0.816789 | H | 4.435533 | 4.336238 | -1.558509 |
| --- | --- | --- | --- | --- | --- | --- | --- |
| C | -2.354012 | -2.316085 | -0.446078 | H | 4.368543 | 3.058565 | -2.767339 |
| C | -2.048517 | -1.082360 | 0.127675 | H | 5.738776 | -2.321866 | 2.940171 |
| C | 0.314062 | -0.034244 | 0.634849 | H | 7.164166 | -2.898275 | 2.051651 |
| C | 1.679927 | -0.198622 | 0.428762 | H | 7.052054 | -1.204345 | 2.516889 |
| C | 2.166310 | -1.342229 | -0.263545 | H | 5.303385 | 1.174817 | 0.411424 |
| C | 1.276734 | -2.324691 | -0.695695 | H | 5.870425 | 1.597266 | -1.211329 |
| C | -3.006035 | -0.091908 | 0.581200 | H | 5.868365 | 2.816076 | 0.055546 |
| C | -2.899383 | 1.248380 | 0.414909 | H | 7.368890 | -0.550856 | -0.000674 |
| C | -3.940324 | 2.138464 | 0.982705 | H | 7.370816 | -2.233783 | -0.516962 |
| N | -3.713896 | 3.489925 | 0.810834 | H | 6.182281 | -1.108132 | -1.192668 |
| C | -2.609791 | 4.101752 | 0.206426 | H | -2.988836 | -5.015255 | -0.841066 |
| C | -1.598003 | 3.195532 | -0.422977 | H | -4.536527 | -4.734988 | -1.639464 |
| N | -1.867643 | 1.857297 | -0.314965 | H | -3.054069 | -4.188499 | -2.427608 |
| C | -3.702653 | -2.939351 | -0.739867 | H | -4.662289 | -1.019012 | -1.204408 |
| C | -4.503836 | -3.190005 | 0.530963 | H | -5.489453 | -2.431823 | -1.890115 |
| C | -4.059131 | -3.156470 | 1.785598 | H | -3.976161 | -1.845986 | -2.613522 |
| C | -4.508516 | -1.995747 | -1.668238 | H | 4.031454 | -0.619432 | -1.024053 |
| C | -3.548954 | -4.301502 | -1.455085 | H | 3.777910 | -2.339024 | -1.244791 |
| C | -2.432379 | 5.428778 | 0.155250 | H | 3.571557 | 0.513557 | 1.204280 |
| C | 2.610800 | 0.915482 | 0.876771 | H | 2.161578 | 1.404159 | 1.752832 |
| C | 2.778710 | 1.940485 | -0.219732 | H | -1.554457 | 5.817291 | -0.343324 |
| C | 3.919104 | 2.394566 | -0.758599 | H | -3.141154 | 6.124213 | 0.593164 |
| C | 5.302948 | 1.962707 | -0.344504 | H | -4.717424 | -3.384716 | 2.618729 |
| C | 3.884223 | 3.432248 | -1.853802 | H | -3.034787 | -2.887000 | 2.025949 |
| C | 3.647484 | -1.518200 | -0.526150 | H | 1.649461 | -3.216398 | -1.193838 |
| C | 4.450885 | -1.804074 | 0.728577 | H | -3.912107 | -0.423032 | 1.077455 |
| C | 5.784091 | -1.742779 | 0.852566 | H | -0.035098 | 0.833509 | 1.185432 |
| C | 6.461634 | -2.060263 | 2.161327 | H | 3.865759 | -2.100717 | 1.598441 |
| C | 6.712890 | -1.386096 | -0.279397 | H | 1.838797 | 2.343267 | -0.599396 |
| C | -0.090211 | -2.132447 | -0.494745 | H | -5.544043 | -3.458125 | 0.341478 |
| C | -0.602049 | -0.970731 | 0.129202 | H | -1.117287 | -3.822437 | -1.278355 |
| O | -4.937764 | 1.735376 | 1.568420 | H | -4.429471 | 4.080924 | 1.217778 |
| O | -0.604592 | 3.611495 | -1.008414 | H | -1.174836 | 1.254505 | -0.745077 |
| H | 2.859540 | 3.723395 | -2.102541 | - | - | - | - |

1. Atomic coordinates (Å) of (8*Z*)-**1**-3 obtained at the B3lyp/6-31G(d) level of theory in the gas phase.

| N | -0.801794 | 2.548486 | 0.510779 | H | 5.304374 | -4.574903 | 1.595497 |
| --- | --- | --- | --- | --- | --- | --- | --- |
| C | -1.923844 | 1.970155 | -0.067011 | H | 5.894067 | -3.137705 | 2.422449 |
| C | -1.536964 | 0.778280 | -0.666542 | H | 7.513206 | 0.710544 | -2.119724 |
| C | 0.827960 | -0.345581 | -0.776698 | H | 6.410717 | 2.073740 | -2.405314 |
| C | 2.155809 | -0.208319 | -0.384681 | H | 7.848652 | 2.273454 | -1.383201 |
| C | 2.561151 | 0.926273 | 0.377867 | H | 6.019310 | -3.637931 | -0.870941 |
| C | 1.633784 | 1.905149 | 0.726578 | H | 5.780299 | -1.901996 | -1.128941 |
| C | -2.322939 | -0.212443 | -1.412925 | H | 6.861035 | -2.478542 | 0.150462 |
| C | -3.158065 | -1.095600 | -0.833768 | H | 6.421945 | 0.074081 | 1.432713 |
| C | -3.891804 | -2.089859 | -1.661954 | H | 7.702634 | -0.248367 | 0.252101 |
| N | -4.746230 | -2.914893 | -0.963416 | H | 7.698310 | 1.267744 | 1.145571 |
| C | -5.000980 | -2.912226 | 0.413858 | H | -4.624265 | 1.165101 | 0.297667 |
| C | -4.239392 | -1.925847 | 1.245642 | H | -5.328790 | 2.622127 | -0.419709 |
| N | -3.381894 | -1.114654 | 0.546600 | H | -4.363731 | 1.517550 | -1.418880 |
| C | -3.202780 | 2.796293 | -0.076655 | H | -2.163182 | 4.531782 | -0.938198 |
| C | -3.394943 | 3.354882 | 1.325536 | H | -3.927476 | 4.553908 | -1.155015 |
| C | -3.343771 | 4.635432 | 1.694938 | H | -2.917611 | 3.460046 | -2.124149 |
| C | -3.041703 | 3.909933 | -1.134879 | H | 4.353088 | 0.201642 | 1.314459 |
| C | -4.452801 | 1.965962 | -0.426639 | H | 4.052050 | 1.916985 | 1.538386 |
| C | -5.871081 | -3.741325 | 1.004803 | H | 4.021364 | -0.852236 | -1.244688 |
| C | 3.149979 | -1.294516 | -0.750493 | H | 2.676618 | -1.951792 | -1.493728 |
| C | 3.585106 | -2.121252 | 0.438247 | H | -6.005756 | -3.677202 | 2.076324 |
| C | 4.771617 | -2.717375 | 0.624439 | H | -6.441660 | -4.475511 | 0.445194 |
| C | 5.910314 | -2.669386 | -0.362465 | H | -3.500813 | 4.926865 | 2.729657 |
| C | 5.040780 | -3.540943 | 1.859044 | H | -3.164703 | 5.440607 | 0.988338 |
| C | 4.006215 | 1.103999 | 0.799745 | H | 1.945524 | 2.769422 | 1.307820 |
| C | 4.925205 | 1.425550 | -0.359134 | H | -2.180587 | -0.324419 | -2.484525 |
| C | 6.240584 | 1.178621 | -0.437612 | H | 0.519975 | -1.218732 | -1.346519 |
| C | 7.036360 | 1.583851 | -1.652841 | H | 4.448039 | 1.932366 | -1.197964 |
| C | 7.044901 | 0.529363 | 0.659392 | H | 2.821629 | -2.252871 | 1.205340 |
| C | 0.308557 | 1.755861 | 0.313564 | H | -3.583693 | 2.593840 | 2.084069 |
| C | -0.113964 | 0.635167 | -0.436683 | H | -0.846926 | 3.385133 | 1.073772 |
| O | -3.765582 | -2.181508 | -2.875124 | H | -5.240896 | -3.583229 | -1.543110 |
| O | -4.364676 | -1.841874 | 2.458914 | H | -2.845418 | -0.456602 | 1.103697 |
| H | 4.174422 | -3.571016 | 2.526972 | - | - | - | - |

1. Conformational analysis of the B3lyp/6-31G(d) optimized conformers of (9*R*)-**2** in the gas phase (T=298.15 K)

| Conformer | ^a^E (Hartree) | ^b^C (Hartree) | ^c^G (kcal/mol) | ΔG (kcal/mol) | Population |
| --- | --- | --- | --- | --- | --- |
| (9*R*)-**2**-1 | -1441.893402 | 0.534677 | -904452.600239 | 0.0 | 65.25% |
| (9*R*)-**2**-2 | -1441.891564 | 0.534545 | -904451.529573 | 1.070666 | 10.70% |
| (9*R*)-**2**-3 | -1441.890314 | 0.533621 | -904451.324845 | 1.275394 | 7.57% |
| (9*R*)-**2**-4 | -1441.889777 | 0.533176 | -904451.267303 | 1.332935 | 6.87% |
| (9*R*)-**2**-5 | -1441.890999 | 0.534669 | -904451.097326 | 1.502913 | 5.15% |
| (9*R*)-**2**-6 | -1441.892792 | 0.536598 | -904451.012049 | 1.58819 | 4.46% |

^a^Electronic energy obtained at M062X/6-311+G(2d,p) level of theory; ^b^Thermal correction to Gibbs free energy obtained at B3lyp/6-31G(d) level of theory; ^c^Gibbs free energy (E + C); The relative Gibbs free energy; The Boltzmann distribution of each conformer.

1. Key transitions, oscillator strengths, and rotatory strengths in the ECD spectrum of conformer (9*R*)-**2**-1 at the B3lyp/6-31G(d) level of theory in MeOH with IEFPCM solvent model.

| Num | transition | CI-coeff | ΔE (eV) | λ (nm) | f | Rvel | Rlen |
| --- | --- | --- | --- | --- | --- | --- | --- |
| 1 | 124->125 | 0.6894 | 4.3948 | 282.11 | 0.0576 | 142.063 | 146.5097 |
| 2 | 123->125 | 0.50808 | 4.7317 | 262.03 | 0.0430 | 5.4674 | 4.5657 |
|  | 123->126 | -0.34476 |  |  |  |  |  |
| 3 | 124->126 | 0.6378 | 4.9247 | 251.76 | 0.1497 | -157.3307 | -158.1029 |
| 4 | 115->125 | 0.24344 | 5.0066 | 247.64 | 0.0063 | 0.1628 | -0.4881 |
|  | 116->125 | 0.33968 |  |  |  |  |  |
|  | 117->125 | -0.24108 |  |  |  |  |  |
|  | 118->125 | -0.33097 |  |  |  |  |  |
|  | 122->125 | -0.22563 |  |  |  |  |  |
| 5 | 123->125 | 0.46441 | 5.1048 | 242.88 | 0.0152 | -14.9101 | -15.5335 |
|  | 123->126 | 0.41474 |  |  |  |  |  |
| 6 | 116->125 | 0.23414 | 5.3890 | 230.07 | 0.0665 | 67.4682 | 69.2913 |
|  | 120->125 | -0.31386 |  |  |  |  |  |
|  | 122->125 | 0.43043 |  |  |  |  |  |
| 7 | 122->125 | 0.2407 | 5.4968 | 225.56 | 0.0159 | -134.3572 | -142.003 |
|  | 124->127 | 0.30262 |  |  |  |  |  |
| 8 | 123->126 | -0.23173 | 5.6502 | 219.43 | 0.3502 | 188.5033 | 190.3499 |
|  | 124->127 | 0.52666 |  |  |  |  |  |
| 9 | 124->128 | 0.30024 | 5.8958 | 210.29 | 0.3218 | 29.4318 | 27.5758 |
|  | 124->129 | 0.48548 |  |  |  |  |  |
| 10 | 119->125 | 0.38777 | 5.9822 | 207.25 | 0.0884 | -65.1162 | -65.2763 |
|  | 121->125 | -0.3407 |  |  |  |  |  |
|  | 124->129 | 0.24369 |  |  |  |  |  |
| 11 | 123->127 | 0.40857 | 6.0386 | 205.32 | 0.2898 | 15.9647 | 11.6633 |
|  | 124->128 | -0.34848 |  |  |  |  |  |
|  | 124->129 | 0.25431 |  |  |  |  |  |
| 12 | 123->127 | 0.25288 | 6.1266 | 202.37 | 0.2206 | 14.1419 | 24.1652 |
|  | 123->128 | -0.29636 |  |  |  |  |  |
|  | 124->128 | 0.32358 |  |  |  |  |  |
| 13 | 120->125 | 0.50071 | 6.1612 | 201.23 | 0.0859 | -35.7508 | -36.1625 |
|  | 122->125 | 0.31573 |  |  |  |  |  |
| 14 | 119->125 | 0.24377 | 6.2218 | 199.27 | 0.0853 | 83.7028 | 92.6531 |
|  | 121->125 | 0.39406 |  |  |  |  |  |
|  | 121->126 | -0.25166 |  |  |  |  |  |
| 15 | 122->126 | 0.276 | 6.4100 | 193.42 | 0.0264 | 46.6921 | 55.2761 |
|  | 122->127 | 0.25722 |  |  |  |  |  |
|  | 123->127 | 0.2767 |  |  |  |  |  |
|  | 123->128 | 0.24401 |  |  |  |  |  |
| 16 | 122->126 | -0.32242 | 6.4313 | 192.78 | 0.0096 | -23.5966 | -23.0062 |
|  | 124->130 | 0.39854 |  |  |  |  |  |
| 17 | 122->126 | 0.42283 | 6.4882 | 191.09 | 0.0364 | -31.271 | -32.0888 |
|  | 123->129 | -0.2916 |  |  |  |  |  |
|  | 124->130 | 0.33469 |  |  |  |  |  |
| 18 | 121->125 | 0.27031 | 6.5607 | 188.98 | 0.2178 | 85.6415 | 86.9014 |
|  | 121->126 | 0.46984 |  |  |  |  |  |
|  | 124->130 | 0.26029 |  |  |  |  |  |
| 19 | 117->125 | 0.44689 | 6.5717 | 188.66 | 0.0971 | -57.712 | -58.7787 |
| 20 | 117->125 | 0.26614 | 6.6302 | 187.00 | 0.0755 | -87.5216 | -91.3979 |
|  | 122->127 | 0.30118 |  |  |  |  |  |
|  | 123->128 | -0.24289 |  |  |  |  |  |
| 21 | 123->128 | -0.31722 | 6.7026 | 184.98 | 0.0189 | 21.8991 | 23.2145 |
|  | 123->129 | 0.34809 |  |  |  |  |  |
| 22 | 120->126 | 0.53583 | 6.7033 | 184.96 | 0.0299 | 26.575 | 23.9997 |
|  | 122->126 | 0.22594 |  |  |  |  |  |
| 23 | 118->125 | 0.35363 | 6.8179 | 181.85 | 0.0725 | -76.8863 | -77.8846 |
|  | 124->131 | 0.36762 |  |  |  |  |  |
| 24 | 115->125 | -0.24435 | 6.8344 | 181.41 | 0.0221 | 14.6736 | 18.1193 |
|  | 118->125 | -0.24149 |  |  |  |  |  |
|  | 124->131 | 0.30433 |  |  |  |  |  |
|  | 124->133 | -0.29974 |  |  |  |  |  |
| 25 | 124->131 | 0.25729 | 6.8807 | 180.19 | 0.0555 | 38.7722 | 40.771 |
|  | 124->133 | 0.39194 |  |  |  |  |  |
| 26 | 119->126 | 0.30933 | 6.9707 | 177.87 | 0.1925 | -17.902 | -20.2773 |
| 27 | 123->130 | 0.46468 | 6.9980 | 177.17 | 0.0353 | -36.1669 | -38.0816 |
| 28 | 116->125 | 0.31761 | 7.0165 | 176.70 | 0.0967 | 53.9936 | 62.3189 |
|  | 119->127 | 0.24879 |  |  |  |  |  |
| 29 | 119->126 | -0.30198 | 7.0523 | 175.81 | 0.0148 | 2.5212 | -0.6034 |
|  | 123->130 | 0.31868 |  |  |  |  |  |
| 30 | 121->127 | -0.24294 | 7.1561 | 173.26 | 0.0884 | -46.2064 | -47.0219 |
|  | 123->131 | 0.32232 |  |  |  |  |  |

Number of the excited states; Only transitions with contribution over 10.0% were listed; Configuration-interaction coefficient; Excitation energy; Wavelength; Rotatory strength in velocity form (10^-40^ cgs); Oscillator strength; Rotatory strength in length form (10^-40^ cgs).

1. Key transitions, oscillator strengths, and rotatory strengths in the ECD spectrum of conformer (9*R*)-**2**-2 at the B3lyp/6-31G(d) level of theory in MeOH with IEFPCM solvent model.

| Num | transition | CI-coeff | ΔE (eV) | λ (nm) | f | Rvel | Rlen |
| --- | --- | --- | --- | --- | --- | --- | --- |
| 1 | 124->125 | 0.68065 | 4.4880 | 276.26 | 0.0490 | 124.6165 | 129.4289 |
| 2 | 123->125 | 0.48743 | 4.7314 | 262.05 | 0.0422 | 6.6788 | 6.6698 |
|  | 123->126 | -0.36139 |  |  |  |  |  |
| 3 | 124->126 | 0.60766 | 4.9911 | 248.41 | 0.1430 | -205.1589 | -206.8354 |
| 4 | 115->125 | 0.25193 | 5.0276 | 246.61 | 0.0152 | 31.6154 | 30.9298 |
|  | 116->125 | 0.32445 |  |  |  |  |  |
|  | 117->125 | -0.28083 |  |  |  |  |  |
|  | 118->125 | -0.24463 |  |  |  |  |  |
|  | 122->125 | -0.237 |  |  |  |  |  |
| 5 | 123->125 | 0.4849 | 5.0993 | 243.14 | 0.0138 | -0.6824 | -0.7807 |
|  | 123->126 | 0.42301 |  |  |  |  |  |
| 6 | 116->125 | 0.26001 | 5.4110 | 229.14 | 0.0629 | 69.3271 | 71.4863 |
|  | 120->125 | 0.30236 |  |  |  |  |  |
|  | 122->125 | 0.40171 |  |  |  |  |  |
| 7 | 119->125 | -0.22429 | 5.5241 | 224.44 | 0.0222 | -129.2559 | -136.3262 |
|  | 122->125 | 0.27151 |  |  |  |  |  |
| 8 | 123->126 | 0.24956 | 5.7194 | 216.78 | 0.3723 | 103.4322 | 100.1139 |
|  | 124->127 | 0.53499 |  |  |  |  |  |
| 9 | 124->127 | -0.24333 | 5.9223 | 209.35 | 0.2233 | -35.8186 | -40.3301 |
|  | 124->128 | 0.42861 |  |  |  |  |  |
|  | 124->129 | 0.33941 |  |  |  |  |  |
| 10 | 124->128 | -0.40512 | 5.9642 | 207.88 | 0.0777 | -63.9122 | -67.2662 |
|  | 124->129 | 0.42328 |  |  |  |  |  |
| 11 | 119->125 | 0.35917 | 6.0208 | 205.93 | 0.0737 | 32.4943 | 39.0654 |
|  | 121->125 | 0.35278 |  |  |  |  |  |
| 12 | 123->127 | 0.413 | 6.0551 | 204.76 | 0.4816 | -70.1311 | -68.3591 |
|  | 123->128 | 0.24822 |  |  |  |  |  |
|  | 123->129 | 0.29635 |  |  |  |  |  |
| 13 | 119->125 | -0.25298 | 6.1809 | 200.59 | 0.0510 | 36.245 | 41.2958 |
|  | 120->125 | 0.48858 |  |  |  |  |  |
|  | 122->125 | -0.34389 |  |  |  |  |  |
| 14 | 119->125 | -0.24431 | 6.2202 | 199.32 | 0.0904 | 120.3991 | 132.4152 |
|  | 120->125 | -0.24805 |  |  |  |  |  |
|  | 121->125 | 0.4047 |  |  |  |  |  |
|  | 121->126 | -0.25756 |  |  |  |  |  |
| 15 | 122->126 | 0.25674 | 6.4165 | 193.23 | 0.0265 | 65.0876 | 74.8476 |
|  | 122->127 | -0.25897 |  |  |  |  |  |
|  | 123->127 | -0.28428 |  |  |  |  |  |
|  | 123->129 | 0.36981 |  |  |  |  |  |
| 16 | 122->126 | -0.28656 | 6.4369 | 192.61 | 0.0009 | -7.1524 | -6.4177 |
|  | 123->127 | -0.22713 |  |  |  |  |  |
|  | 124->130 | 0.38087 |  |  |  |  |  |
| 17 | 122->126 | 0.46926 | 6.4850 | 191.18 | 0.0223 | -45.1363 | -44.3009 |
|  | 123->129 | -0.22753 |  |  |  |  |  |
|  | 124->130 | 0.34053 |  |  |  |  |  |
| 18 | 121->125 | 0.24289 | 6.5465 | 189.39 | 0.2385 | 46.7145 | 46.2706 |
|  | 121->126 | 0.48796 |  |  |  |  |  |
|  | 124->130 | 0.2965 |  |  |  |  |  |
| 19 | 117->125 | 0.2782 | 6.5779 | 188.49 | 0.1248 | -75.3849 | -77.5532 |
|  | 122->127 | 0.30871 |  |  |  |  |  |
| 20 | 117->125 | 0.3724 | 6.6463 | 186.55 | 0.0322 | -54.6572 | -55.7347 |
| 21 | 123->128 | 0.50897 | 6.6844 | 185.48 | 0.0205 | 26.7283 | 25.5518 |
| 22 | 120->126 | 0.53292 | 6.7017 | 185.01 | 0.0133 | 20.166 | 17.9084 |
| 23 | 123->130 | 0.4701 | 6.7883 | 182.64 | 0.0526 | 2.0769 | 4.253 |
|  | 124->133 | -0.25429 |  |  |  |  |  |
| 24 | 123->131 | 0.24936 | 6.8287 | 181.56 | 0.0309 | 28.351 | 28.4263 |
|  | 124->131 | 0.47265 |  |  |  |  |  |
| 25 | 115->125 | 0.37394 | 6.9069 | 179.51 | 0.0025 | -1.6514 | -1.0746 |
|  | 118->125 | 0.38527 |  |  |  |  |  |
|  | 119->127 | -0.22887 |  |  |  |  |  |
| 26 | 122->129 | -0.25258 | 6.9501 | 178.39 | 0.1300 | -20.5064 | -25.1111 |
|  | 124->132 | 0.28411 |  |  |  |  |  |
|  | 124->133 | 0.25268 |  |  |  |  |  |
| 27 | 123->130 | 0.32128 | 7.0015 | 177.08 | 0.2609 | 8.7383 | 10.0939 |
|  | 124->132 | 0.31179 |  |  |  |  |  |
|  | 124->133 | 0.23736 |  |  |  |  |  |
| 28 | 117->126 | -0.25869 | 7.0633 | 175.53 | 0.0176 | 23.6466 | 24.3233 |
|  | 119->126 | 0.33257 |  |  |  |  |  |
|  | 123->130 | 0.23389 |  |  |  |  |  |
| 29 | 116->125 | 0.37036 | 7.0800 | 175.12 | 0.0286 | 13.0588 | 19.1638 |
|  | 118->125 | 0.3369 |  |  |  |  |  |
|  | 119->127 | 0.30985 |  |  |  |  |  |
| 30 | 123->133 | 0.33039 | 7.0979 | 174.68 | 0.0387 | 66.1059 | 68.8639 |

Number of the excited states; Only transitions with contribution over 10.0% were listed; Configuration-interaction coefficient; Excitation energy; Wavelength; Rotatory strength in velocity form (10^-40^ cgs); Oscillator strength; Rotatory strength in length form (10^-40^ cgs).

1. Key transitions, oscillator strengths, and rotatory strengths in the ECD spectrum of conformer (9*R*)-**2**-3 at the B3lyp/6-31G(d) level of theory in MeOH with IEFPCM solvent model.

| Num | transition | CI-coeff | ΔE (eV) | λ (nm) | f | Rvel | Rlen |
| --- | --- | --- | --- | --- | --- | --- | --- |
| 1 | 124->125 | 0.68021 | 4.4742 | 277.11 | 0.0614 | 98.5288 | 103.3104 |
| 2 | 123->125 | 0.46639 | 4.7337 | 261.92 | 0.0419 | -5.0249 | -4.1091 |
|  | 123->126 | -0.37204 |  |  |  |  |  |
| 3 | 124->126 | 0.615 | 4.9677 | 249.58 | 0.1566 | -153.9345 | -155.0377 |
| 4 | 116->125 | 0.46869 | 5.0324 | 246.37 | 0.0130 | 13.8948 | 12.6991 |
|  | 122->125 | -0.25328 |  |  |  |  |  |
| 5 | 123->125 | 0.49784 | 5.0818 | 243.98 | 0.0098 | 0.8184 | 0.9712 |
|  | 123->126 | 0.41392 |  |  |  |  |  |
| 6 | 116->125 | 0.32759 | 5.4145 | 228.99 | 0.0471 | 80.9096 | 83.0492 |
|  | 120->125 | -0.29923 |  |  |  |  |  |
|  | 122->125 | 0.28908 |  |  |  |  |  |
| 7 | 120->125 | -0.24567 | 5.5239 | 224.45 | 0.0443 | -147.7679 | -155.6325 |
|  | 122->125 | 0.30559 |  |  |  |  |  |
| 8 | 123->126 | 0.26708 | 5.7204 | 216.74 | 0.4777 | 140.3868 | 140.5173 |
|  | 124->127 | 0.52828 |  |  |  |  |  |
| 9 | 124->128 | -0.23925 | 5.9238 | 209.30 | 0.2790 | -62.2713 | -65.924 |
|  | 124->129 | 0.50994 |  |  |  |  |  |
| 10 | 115->125 | 0.22467 | 6.0329 | 205.51 | 0.0537 | 25.5107 | 33.0545 |
|  | 119->125 | 0.4091 |  |  |  |  |  |
| 11 | 123->127 | 0.43981 | 6.0873 | 203.68 | 0.4593 | -216.2504 | -223.0412 |
|  | 123->129 | 0.33835 |  |  |  |  |  |
| 12 | 124->128 | 0.51712 | 6.1390 | 201.96 | 0.0755 | 33.2065 | 34.0687 |
| 13 | 119->125 | 0.22815 | 6.1870 | 200.39 | 0.0240 | 0.8522 | 3.6517 |
|  | 120->125 | 0.43521 |  |  |  |  |  |
|  | 122->125 | 0.39659 |  |  |  |  |  |
| 14 | 117->125 | 0.23278 | 6.2762 | 197.55 | 0.0704 | 126.507 | 139.248 |
|  | 121->125 | -0.34757 |  |  |  |  |  |
|  | 121->126 | 0.35969 |  |  |  |  |  |
| 15 | 122->127 | -0.22845 | 6.4360 | 192.64 | 0.0296 | 69.9167 | 79.185 |
|  | 123->127 | -0.2896 |  |  |  |  |  |
|  | 123->129 | 0.38507 |  |  |  |  |  |
| 16 | 123->128 | 0.49636 | 6.4708 | 191.61 | 0.0224 | 25.9071 | 28.3224 |
|  | 124->130 | 0.32037 |  |  |  |  |  |
| 17 | 122->126 | 0.51295 | 6.4845 | 191.20 | 0.0238 | -23.2319 | -25.003 |
|  | 123->127 | 0.23299 |  |  |  |  |  |
|  | 123->129 | -0.23137 |  |  |  |  |  |
| 18 | 117->125 | -0.37655 | 6.5621 | 188.94 | 0.0828 | -75.3901 | -78.2426 |
|  | 121->126 | 0.3885 |  |  |  |  |  |
| 19 | 124->130 | 0.41713 | 6.6065 | 187.67 | 0.0560 | 15.7863 | 17.1092 |
| 20 | 123->128 | 0.33849 | 6.6300 | 187.01 | 0.0429 | 115.7105 | 120.5539 |
|  | 124->130 | -0.32716 |  |  |  |  |  |
| 21 | 120->126 | 0.33003 | 6.6451 | 186.58 | 0.1071 | -82.1345 | -83.5015 |
|  | 122->126 | 0.22795 |  |  |  |  |  |
| 22 | 117->125 | -0.23457 | 6.7033 | 184.96 | 0.0123 | 19.9847 | 16.5547 |
|  | 120->126 | 0.40849 |  |  |  |  |  |
| 23 | 124->131 | 0.43637 | 6.8394 | 181.28 | 0.1479 | -23.952 | -22.9575 |
|  | 124->132 | -0.25884 |  |  |  |  |  |
| 24 | 123->131 | 0.28777 | 6.9093 | 179.45 | 0.0909 | -40.0884 | -41.3773 |
|  | 124->132 | 0.38607 |  |  |  |  |  |
| 25 | 120->127 | 0.2514 | 6.9748 | 177.76 | 0.1639 | -7.046 | -10.3399 |
|  | 122->127 | 0.24725 |  |  |  |  |  |
|  | 122->129 | 0.28764 |  |  |  |  |  |
|  | 123->130 | 0.22924 |  |  |  |  |  |
| 26 | 123->130 | 0.51801 | 6.9795 | 177.64 | 0.0641 | -28.6543 | -27.7502 |
| 27 | 115->125 | 0.41542 | 7.0135 | 176.78 | 0.0264 | 29.8753 | 35.2156 |
|  | 116->125 | -0.23734 |  |  |  |  |  |
|  | 119->127 | -0.30817 |  |  |  |  |  |
| 28 | 117->126 | 0.22824 | 7.0681 | 175.41 | 0.0262 | 46.646 | 48.1434 |
|  | 119->126 | 0.34395 |  |  |  |  |  |
| 29 | 118->125 | 0.23858 | 7.0889 | 174.90 | 0.0467 | -20.8735 | -26.154 |
|  | 121->128 | -0.23524 |  |  |  |  |  |
| 30 | 121->127 | -0.25097 | 7.1445 | 173.54 | 0.1889 | -24.5959 | -21.239 |
|  | 121->128 | 0.27646 |  |  |  |  |  |

Number of the excited states; Only transitions with contribution over 10.0% were listed; Configuration-interaction coefficient; Excitation energy; Wavelength; Rotatory strength in velocity form (10^-40^ cgs); Oscillator strength; Rotatory strength in length form (10^-40^ cgs).

1. Key transitions, oscillator strengths, and rotatory strengths in the ECD spectrum of conformer (9*R*)-**2**-4 at the B3lyp/6-31G(d) level of theory in MeOH with IEFPCM solvent model.

| Num | transition | CI-coeff | ΔE (eV) | λ (nm) | f | Rvel | Rlen |
| --- | --- | --- | --- | --- | --- | --- | --- |
| 1 | 124->125 | 0.68887 | 4.3848 | 282.76 | 0.0549 | 120.6989 | 123.1634 |
| 2 | 123->125 | 0.49843 | 4.7362 | 261.78 | 0.0365 | -1.093 | -1.5044 |
|  | 123->126 | -0.35 |  |  |  |  |  |
| 3 | 124->126 | 0.63194 | 4.9280 | 251.59 | 0.1297 | -118.4722 | -119.4354 |
| 4 | 116->125 | 0.45681 | 5.0083 | 247.56 | 0.0065 | -5.0478 | -5.9824 |
|  | 117->125 | -0.23625 |  |  |  |  |  |
|  | 122->125 | -0.24229 |  |  |  |  |  |
| 5 | 123->125 | 0.47369 | 5.1004 | 243.09 | 0.0164 | -8.913 | -9.3753 |
|  | 123->126 | 0.41968 |  |  |  |  |  |
| 6 | 116->125 | 0.28657 | 5.3869 | 230.16 | 0.0616 | 73.4151 | 75.3427 |
|  | 120->125 | -0.30804 |  |  |  |  |  |
|  | 122->125 | 0.39834 |  |  |  |  |  |
| 7 | 119->125 | -0.22491 | 5.5038 | 225.27 | 0.0250 | -131.7908 | -138.9533 |
|  | 122->125 | 0.29404 |  |  |  |  |  |
| 8 | 123->126 | 0.23775 | 5.7031 | 217.40 | 0.2877 | 111.2405 | 108.6113 |
|  | 124->127 | 0.57475 |  |  |  |  |  |
| 9 | 124->128 | 0.55262 | 5.9012 | 210.10 | 0.3909 | -35.8477 | -40.0477 |
| 10 | 119->125 | 0.31517 | 5.9986 | 206.69 | 0.0840 | -13.0626 | -10.6123 |
|  | 121->125 | 0.35675 |  |  |  |  |  |
| 11 | 123->127 | 0.46322 | 6.1003 | 203.24 | 0.4608 | -49.6234 | -46.7434 |
|  | 123->128 | 0.31957 |  |  |  |  |  |
| 12 | 124->129 | 0.61484 | 6.1216 | 202.53 | 0.0143 | 38.4662 | 38.7363 |
| 13 | 120->125 | 0.49913 | 6.1702 | 200.94 | 0.0447 | 18.7251 | 22.1557 |
|  | 122->125 | 0.33118 |  |  |  |  |  |
| 14 | 119->125 | -0.2513 | 6.2304 | 199.00 | 0.0882 | 91.4789 | 102.5391 |
|  | 121->125 | 0.39062 |  |  |  |  |  |
|  | 121->126 | -0.26226 |  |  |  |  |  |
| 15 | 124->130 | 0.54253 | 6.4021 | 193.66 | 0.0218 | -38.9767 | -38.531 |
| 16 | 122->126 | 0.31739 | 6.4355 | 192.66 | 0.0296 | 31.189 | 37.9185 |
|  | 122->127 | -0.28552 |  |  |  |  |  |
|  | 123->127 | -0.24759 |  |  |  |  |  |
|  | 123->128 | 0.31332 |  |  |  |  |  |
| 17 | 122->126 | 0.49489 | 6.4945 | 190.91 | 0.0471 | -23.2737 | -24.8043 |
|  | 123->127 | 0.24861 |  |  |  |  |  |
|  | 123->128 | -0.31616 |  |  |  |  |  |
| 18 | 117->125 | -0.39039 | 6.5501 | 189.28 | 0.1872 | 1.0743 | -1.7662 |
|  | 118->125 | 0.25298 |  |  |  |  |  |
|  | 121->126 | 0.42306 |  |  |  |  |  |
| 19 | 121->125 | 0.32015 | 6.5609 | 188.97 | 0.0847 | 75.2421 | 83.2974 |
|  | 121->126 | 0.3014 |  |  |  |  |  |
| 20 | 122->127 | 0.35292 | 6.6456 | 186.57 | 0.0955 | -82.8337 | -86.9673 |
|  | 123->128 | 0.27515 |  |  |  |  |  |
| 21 | 120->126 | 0.57863 | 6.7091 | 184.80 | 0.0294 | 22.1828 | 18.2527 |
| 22 | 123->130 | 0.38424 | 6.7663 | 183.24 | 0.0352 | 33.7233 | 34.5563 |
|  | 124->131 | -0.25699 |  |  |  |  |  |
|  | 124->133 | -0.29431 |  |  |  |  |  |
| 23 | 123->131 | 0.27711 | 6.8138 | 181.96 | 0.0408 | -3.8461 | -3.7814 |
|  | 124->131 | 0.45944 |  |  |  |  |  |
| 24 | 123->129 | 0.43386 | 6.8971 | 179.76 | 0.0629 | -1.6968 | -3.2555 |
|  | 124->132 | -0.23957 |  |  |  |  |  |
|  | 124->133 | -0.29401 |  |  |  |  |  |
| 25 | 115->125 | 0.30059 | 6.9362 | 178.75 | 0.0118 | -18.1298 | -19.7041 |
|  | 118->125 | 0.32707 |  |  |  |  |  |
|  | 119->125 | -0.29222 |  |  |  |  |  |
| 26 | 123->129 | 0.41064 | 6.9561 | 178.24 | 0.0750 | -7.5607 | -10.8598 |
| 27 | 123->130 | 0.33045 | 6.9981 | 177.17 | 0.2012 | -86.161 | -91.2056 |
| 28 | 115->125 | 0.29476 | 7.0174 | 176.68 | 0.0677 | 75.7259 | 87.8992 |
|  | 116->125 | -0.24582 |  |  |  |  |  |
|  | 119->127 | -0.26638 |  |  |  |  |  |
| 29 | 117->126 | -0.25087 | 7.0623 | 175.56 | 0.0189 | 29.2663 | 28.8403 |
|  | 118->126 | 0.28403 |  |  |  |  |  |
|  | 119->126 | 0.2381 |  |  |  |  |  |
| 30 | 123->133 | 0.27866 | 7.1267 | 173.97 | 0.0939 | 27.973 | 31.8156 |
|  | 124->132 | 0.29323 |  |  |  |  |  |

Number of the excited states; Only transitions with contribution over 10.0% were listed; Configuration-interaction coefficient; Excitation energy; Wavelength; Rotatory strength in velocity form (10^-40^ cgs); Oscillator strength; Rotatory strength in length form (10^-40^ cgs).

1. Key transitions, oscillator strengths, and rotatory strengths in the ECD spectrum of conformer (9*R*)-**2**-5 at the B3lyp/6-31G(d) level of theory in MeOH with IEFPCM solvent model.

| Num | transition | CI-coeff | ΔE (eV) | λ (nm) | f | Rvel | Rlen |
| --- | --- | --- | --- | --- | --- | --- | --- |
| 1 | 124->125 | 0.68463 | 4.4228 | 280.33 | 0.0450 | 115.1641 | 119.6451 |
| 2 | 123->125 | 0.54078 | 4.6804 | 264.90 | 0.0386 | -9.868 | -9.9302 |
|  | 123->126 | -0.31547 |  |  |  |  |  |
| 3 | 124->126 | 0.61618 | 4.9819 | 248.87 | 0.1477 | -196.347 | -198.1092 |
| 4 | 116->125 | 0.4408 | 5.0193 | 247.01 | 0.0222 | 41.5775 | 40.4206 |
| 5 | 123->125 | 0.39411 | 5.0520 | 245.42 | 0.0108 | 4.8865 | 5.9425 |
|  | 123->126 | 0.45881 |  |  |  |  |  |
| 6 | 116->125 | 0.31602 | 5.3905 | 230.01 | 0.0598 | 72.9161 | 74.8381 |
|  | 120->125 | 0.33517 |  |  |  |  |  |
|  | 122->125 | 0.3281 |  |  |  |  |  |
| 7 | 119->125 | 0.24893 | 5.5074 | 225.12 | 0.0243 | -135.3988 | -143.1221 |
|  | 122->125 | -0.24164 |  |  |  |  |  |
| 8 | 123->126 | 0.2606 | 5.7025 | 217.42 | 0.4171 | 48.8726 | 45.9269 |
|  | 124->127 | 0.54485 |  |  |  |  |  |
| 9 | 119->125 | -0.24844 | 5.9178 | 209.51 | 0.3108 | -185.8727 | -194.4216 |
|  | 124->128 | 0.3695 |  |  |  |  |  |
|  | 124->129 | 0.37316 |  |  |  |  |  |
| 10 | 121->125 | 0.36153 | 5.9697 | 207.69 | 0.0624 | -38.4816 | -38.4181 |
|  | 122->125 | -0.2952 |  |  |  |  |  |
|  | 124->129 | 0.25958 |  |  |  |  |  |
| 11 | 123->127 | 0.48707 | 6.0405 | 205.26 | 0.3843 | 52.0242 | 62.5263 |
|  | 123->129 | -0.2578 |  |  |  |  |  |
| 12 | 124->128 | 0.43295 | 6.1186 | 202.64 | 0.0289 | -7.7609 | -7.1235 |
|  | 124->129 | -0.31037 |  |  |  |  |  |
| 13 | 120->125 | 0.47928 | 6.1483 | 201.66 | 0.0033 | 4.5975 | 5.5138 |
|  | 121->125 | -0.36354 |  |  |  |  |  |
|  | 122->125 | -0.23106 |  |  |  |  |  |
| 14 | 115->125 | 0.26268 | 6.1788 | 200.66 | 0.1603 | 56.7152 | 67.1848 |
|  | 119->125 | 0.39356 |  |  |  |  |  |
|  | 124->128 | 0.26252 |  |  |  |  |  |
| 15 | 123->127 | 0.29823 | 6.4021 | 193.66 | 0.0165 | 101.7837 | 112.4147 |
|  | 123->128 | 0.41023 |  |  |  |  |  |
|  | 123->129 | 0.27384 |  |  |  |  |  |
| 16 | 120->126 | 0.23603 | 6.4200 | 193.12 | 0.0808 | -54.5925 | -56.6529 |
|  | 121->126 | 0.39872 |  |  |  |  |  |
| 17 | 122->126 | 0.54822 | 6.4699 | 191.63 | 0.0287 | 31.609 | 34.6409 |
|  | 123->129 | 0.27123 |  |  |  |  |  |
| 18 | 121->126 | 0.3632 | 6.5054 | 190.59 | 0.0554 | 94.1496 | 91.802 |
|  | 123->128 | -0.35418 |  |  |  |  |  |
|  | 123->129 | 0.23753 |  |  |  |  |  |
| 19 | 117->125 | 0.49914 | 6.5425 | 189.51 | 0.1071 | -40.9757 | -36.1581 |
| 20 | 122->127 | 0.29741 | 6.6192 | 187.31 | 0.0463 | -55.6865 | -56.8983 |
| 21 | 120->126 | 0.5067 | 6.6924 | 185.26 | 0.0107 | 29.1053 | 27.9451 |
|  | 122->126 | -0.25476 |  |  |  |  |  |
| 22 | 124->130 | 0.53214 | 6.7081 | 184.83 | 0.1065 | -102.8953 | -109.4845 |
| 23 | 123->131 | 0.29347 | 6.8146 | 181.94 | 0.0350 | 83.3601 | 84.3194 |
|  | 124->131 | 0.48703 |  |  |  |  |  |
| 24 | 123->130 | 0.34166 | 6.9196 | 179.18 | 0.0962 | 47.2805 | 49.7745 |
|  | 124->132 | -0.23054 |  |  |  |  |  |
|  | 124->135 | 0.28896 |  |  |  |  |  |
| 25 | 118->125 | 0.57954 | 6.9310 | 178.88 | 0.0500 | -45.6887 | -46.8771 |
| 26 | 120->127 | -0.23912 | 6.9543 | 178.28 | 0.1447 | 113.0984 | 112.4659 |
|  | 122->127 | 0.26887 |  |  |  |  |  |
| 27 | 115->125 | 0.42592 | 6.9902 | 177.37 | 0.0025 | -22.7776 | -16.9599 |
|  | 116->125 | -0.262 |  |  |  |  |  |
|  | 119->127 | -0.29249 |  |  |  |  |  |
| 28 | 117->126 | 0.26379 | 7.0479 | 175.92 | 0.1170 | 27.1505 | 26.556 |
|  | 119->126 | 0.36543 |  |  |  |  |  |
|  | 120->127 | 0.22947 |  |  |  |  |  |
| 29 | 121->128 | 0.39358 | 7.0760 | 175.22 | 0.1453 | -56.7621 | -55.175 |
| 30 | 123->130 | 0.50548 | 7.1226 | 174.07 | 0.0789 | 34.8815 | 36.0663 |
|  | 124->132 | 0.28277 |  |  |  |  |  |

Number of the excited states; Only transitions with contribution over 10.0% were listed; Configuration-interaction coefficient; Excitation energy; Wavelength; Rotatory strength in velocity form (10^-40^ cgs); Oscillator strength; Rotatory strength in length form (10^-40^ cgs).

1. Key transitions, oscillator strengths, and rotatory strengths in the ECD spectrum of conformer (9*R*)-**2**-6 at the B3lyp/6-31G(d) level of theory in MeOH with IEFPCM solvent model.

| Num | transition | CI-coeff | ΔE (eV) | λ (nm) | f | Rvel | Rlen |
| --- | --- | --- | --- | --- | --- | --- | --- |
| 1 | 124->125 | 0.68744 | 4.4298 | 279.89 | 0.0549 | 122.5696 | 126.5284 |
| 2 | 123->125 | -0.35923 | 4.7868 | 259.01 | 0.0417 | -2.8992 | -3.2485 |
|  | 123->126 | 0.37363 |  |  |  |  |  |
|  | 124->126 | 0.3425 |  |  |  |  |  |
| 3 | 123->126 | -0.26808 | 4.8837 | 253.88 | 0.1546 | -83.2047 | -81.4086 |
|  | 124->126 | 0.56307 |  |  |  |  |  |
| 4 | 115->125 | 0.35241 | 5.0109 | 247.43 | 0.0006 | 3.8793 | 4.7111 |
|  | 116->125 | 0.28075 |  |  |  |  |  |
|  | 117->125 | -0.3259 |  |  |  |  |  |
|  | 118->125 | -0.30572 |  |  |  |  |  |
| 5 | 123->125 | 0.57803 | 5.1338 | 241.51 | 0.0060 | -11.0103 | -11.3082 |
|  | 123->126 | 0.3065 |  |  |  |  |  |
| 6 | 120->125 | 0.3578 | 5.3503 | 231.73 | 0.0983 | -37.5692 | -35.5565 |
|  | 121->125 | 0.50126 |  |  |  |  |  |
|  | 122->125 | 0.24261 |  |  |  |  |  |
| 7 | 116->125 | -0.23303 | 5.4729 | 226.54 | 0.0431 | -92.0446 | -99.2761 |
|  | 116->127 | -0.27623 |  |  |  |  |  |
|  | 124->127 | 0.3793 |  |  |  |  |  |
| 8 | 123->126 | -0.22689 | 5.6203 | 220.60 | 0.3043 | 118.9922 | 119.3856 |
|  | 124->127 | 0.47179 |  |  |  |  |  |
| 9 | 124->128 | 0.56016 | 5.8756 | 211.02 | 0.3507 | -78.6779 | -82.1551 |
| 10 | 115->125 | -0.23326 | 6.0050 | 206.47 | 0.0727 | -2.6738 | -0.6621 |
|  | 119->125 | 0.4533 |  |  |  |  |  |
| 11 | 123->127 | -0.36656 | 6.0599 | 204.60 | 0.3000 | 145.6921 | 140.8062 |
|  | 124->129 | 0.43446 |  |  |  |  |  |
| 12 | 121->125 | -0.27946 | 6.0836 | 203.80 | 0.0182 | 54.6886 | 65.2895 |
|  | 122->125 | 0.44609 |  |  |  |  |  |
| 13 | 120->125 | 0.53066 | 6.1413 | 201.89 | 0.0415 | -13.6226 | -12.4074 |
|  | 121->125 | -0.25242 |  |  |  |  |  |
| 14 | 123->127 | 0.36965 | 6.1648 | 201.12 | 0.1961 | -82.3898 | -84.7096 |
|  | 124->129 | 0.32942 |  |  |  |  |  |
| 15 | 124->130 | 0.62755 | 6.2107 | 199.63 | 0.0592 | -30.3389 | -32.559 |
| 16 | 122->125 | 0.26185 | 6.4119 | 193.36 | 0.1110 | -68.3893 | -73.9414 |
|  | 122->126 | 0.55207 |  |  |  |  |  |
| 17 | 121->126 | 0.29997 | 6.4414 | 192.48 | 0.0851 | 165.124 | 182.0243 |
|  | 121->127 | 0.35753 |  |  |  |  |  |
| 18 | 123->127 | 0.39268 | 6.5100 | 190.45 | 0.0784 | -80.2829 | -84.5391 |
|  | 123->128 | 0.42477 |  |  |  |  |  |
|  | 123->129 | 0.2522 |  |  |  |  |  |
| 19 | 117->125 | 0.43442 | 6.5856 | 188.26 | 0.0048 | -41.4367 | -34.9361 |
| 20 | 121->126 | 0.55 | 6.6282 | 187.05 | 0.0212 | -9.5591 | -10.3936 |
|  | 121->127 | -0.26767 |  |  |  |  |  |
| 21 | 123->130 | 0.58833 | 6.6793 | 185.63 | 0.0156 | -18.7544 | -20.3796 |
| 22 | 120->126 | 0.5867 | 6.7160 | 184.61 | 0.0241 | 35.8615 | 36.675 |
| 23 | 123->128 | -0.27583 | 6.7379 | 184.01 | 0.0169 | -27.0208 | -28.9137 |
|  | 123->130 | 0.22873 |  |  |  |  |  |
|  | 124->131 | 0.33289 |  |  |  |  |  |
| 24 | 123->129 | 0.32836 | 6.7876 | 182.66 | 0.0407 | -18.9648 | -18.7998 |
|  | 124->131 | -0.32619 |  |  |  |  |  |
| 25 | 115->125 | 0.26704 | 6.8324 | 181.46 | 0.0577 | -25.1055 | -24.1421 |
|  | 118->125 | 0.29722 |  |  |  |  |  |
|  | 124->133 | 0.34375 |  |  |  |  |  |
| 26 | 118->125 | -0.26223 | 6.8549 | 180.87 | 0.0739 | 88.4323 | 90.5253 |
|  | 124->131 | -0.28069 |  |  |  |  |  |
|  | 124->133 | 0.31049 |  |  |  |  |  |
| 27 | 119->126 | -0.23093 | 6.8723 | 180.41 | 0.2352 | -46.4079 | -66.6502 |
|  | 122->130 | 0.32366 |  |  |  |  |  |
| 28 | 119->126 | 0.26775 | 6.9760 | 177.73 | 0.2658 | 15.9788 | 6.5337 |
|  | 122->130 | 0.41182 |  |  |  |  |  |
| 29 | 116->125 | 0.3724 | 7.0250 | 176.49 | 0.1076 | 126.0146 | 133.9702 |
|  | 118->125 | 0.2335 |  |  |  |  |  |
|  | 119->127 | 0.25422 |  |  |  |  |  |
| 30 | 116->125 | -0.24708 | 7.0496 | 175.87 | 0.0427 | -38.0331 | -41.3563 |
|  | 120->127 | 0.33062 |  |  |  |  |  |

Number of the excited states; Only transitions with contribution over 10.0% were listed; Configuration-interaction coefficient; Excitation energy; Wavelength; Rotatory strength in velocity form (10^-40^ cgs); Oscillator strength; Rotatory strength in length form (10^-40^ cgs).

1. Conformational analysis of the B3lyp/6-31G(d) optimized conformers of (12*S*28*S*31*R*)-**3** in the gas phase (T=298.15 K)

| Conformer | E (Hartree) | C (Hartree) | G (kcal/mol) | ΔG (kcal/mol) | Population |
| --- | --- | --- | --- | --- | --- |
| (12*S*28*S*31*R*)-**3**-1 | -2012.89998 | 0.639006 | -1262693.761254 | 0.0 | 35.94% |
| (12*S*28*S*31*R*)-**3**-2 | -2012.898053 | 0.637304 | -1262693.619935 | 0.141319 | 28.31% |
| (12*S*28*S*31*R*)-**3**-3 | -2012.898339 | 0.637957 | -1262693.389887 | 0.371367 | 19.19% |
| (12*S*28*S*31*R*)-**3**-4 | -2012.897967 | 0.637724 | -1262693.302727 | 0.458527 | 16.57% |

Electronic energy obtained at M062X/6-311+G(2d,p) level of theory; Thermal correction to Gibbs free energy obtained at @@@ level of theory; Gibbs free energy (E + C); The relative Gibbs free energy; The Boltzmann distribution of each conformer.

1. Key transitions, oscillator strengths, and rotatory strengths in the ECD spectrum of conformer (12*S*28*S*31*R*)-**3**-1 at the B3lyp/6-31G(d) level of theory in MeOH with IEFPCM solvent model.

| Num | transition | CI-coeff | ΔE (eV) | λ (nm) | f | Rvel | Rlen |
| --- | --- | --- | --- | --- | --- | --- | --- |
| 1 | 164->166 | 0.66951 | 3.5180 | 352.43 | 0.0993 | -33.096 | -48.347 |
| 2 | 165->166 | 0.26239 | 3.8167 | 324.85 | 0.5416 | 11.8368 | 14.9885 |
|  | 165->167 | 0.61422 |  |  |  |  |  |
| 3 | 165->166 | 0.62367 | 4.0024 | 309.77 | 0.0283 | 23.011 | 22.7528 |
|  | 165->167 | -0.26712 |  |  |  |  |  |
| 4 | 156->166 | 0.44262 | 4.0757 | 304.20 | 0.0083 | 27.0123 | 29.3314 |
|  | 157->166 | 0.30475 |  |  |  |  |  |
| 5 | 164->167 | 0.65572 | 4.4302 | 279.86 | 0.0284 | 5.2798 | 3.6142 |
| 6 | 160->166 | -0.28873 | 4.6105 | 268.91 | 0.0950 | -93.2476 | -88.4189 |
|  | 161->166 | 0.37516 |  |  |  |  |  |
|  | 164->168 | -0.30332 |  |  |  |  |  |
| 7 | 163->167 | -0.3053 | 4.7828 | 259.23 | 0.0215 | -11.8068 | -7.9433 |
|  | 165->169 | 0.55293 |  |  |  |  |  |
| 8 | 163->166 | 0.4089 | 4.8454 | 255.88 | 0.0918 | 59.3573 | 59.7491 |
|  | 163->167 | 0.23507 |  |  |  |  |  |
|  | 163->169 | -0.24478 |  |  |  |  |  |
|  | 165->169 | 0.3129 |  |  |  |  |  |
| 9 | 163->166 | 0.52771 | 4.9465 | 250.65 | 0.0294 | 76.3199 | 79.7268 |
|  | 163->167 | -0.31323 |  |  |  |  |  |
| 10 | 161->166 | 0.23965 | 5.0066 | 247.64 | 0.1089 | -145.6125 | -148.7756 |
|  | 164->168 | 0.55592 |  |  |  |  |  |
| 11 | 154->167 | 0.28192 | 5.0581 | 245.12 | 0.0116 | 18.421 | 20.9865 |
|  | 155->167 | -0.2696 |  |  |  |  |  |
|  | 160->167 | -0.24017 |  |  |  |  |  |
| 12 | 161->166 | -0.30459 | 5.1190 | 242.20 | 0.0113 | -4.4053 | -5.6022 |
|  | 162->166 | 0.60696 |  |  |  |  |  |
| 13 | 160->166 | 0.35915 | 5.1707 | 239.78 | 0.0213 | -3.4879 | -5.4223 |
|  | 165->168 | 0.49523 |  |  |  |  |  |
| 14 | 161->167 | 0.32445 | 5.2904 | 234.36 | 0.0049 | 7.7473 | 10.2969 |
|  | 163->167 | 0.2343 |  |  |  |  |  |
|  | 163->169 | 0.34071 |  |  |  |  |  |
|  | 165->173 | 0.25459 |  |  |  |  |  |
| 15 | 161->167 | -0.31012 | 5.3337 | 232.45 | 0.0592 | 33.6366 | 35.0885 |
|  | 163->167 | 0.38337 |  |  |  |  |  |
| 16 | 160->166 | 0.41172 | 5.4123 | 229.08 | 0.0143 | 15.4763 | 16.3587 |
|  | 161->166 | 0.30537 |  |  |  |  |  |
|  | 165->168 | -0.34383 |  |  |  |  |  |
| 17 | 155->167 | 0.2951 | 5.5228 | 224.49 | 0.0062 | -49.8636 | -51.5421 |
|  | 155->170 | 0.29574 |  |  |  |  |  |
|  | 155->171 | -0.23434 |  |  |  |  |  |
| 18 | 160->167 | 0.31247 | 5.6348 | 220.03 | 0.0680 | 104.0925 | 109.1768 |
|  | 165->170 | -0.2811 |  |  |  |  |  |
| 19 | 160->167 | 0.28855 | 5.7205 | 216.74 | 0.1672 | -80.7858 | -84.7058 |
|  | 165->170 | 0.4339 |  |  |  |  |  |
|  | 165->171 | -0.22556 |  |  |  |  |  |
| 20 | 162->167 | 0.27662 | 5.7368 | 216.12 | 0.0086 | 16.7647 | 15.7176 |
|  | 164->169 | 0.60639 |  |  |  |  |  |
| 21 | 159->166 | 0.35803 | 5.7647 | 215.08 | 0.0790 | 63.5045 | 61.1849 |
|  | 160->167 | -0.24039 |  |  |  |  |  |
|  | 162->167 | 0.25704 |  |  |  |  |  |
| 22 | 159->166 | 0.28186 | 5.8523 | 211.86 | 0.2263 | -52.9886 | -57.6788 |
|  | 160->168 | -0.24801 |  |  |  |  |  |
|  | 161->168 | 0.29388 |  |  |  |  |  |
| 23 | 160->167 | 0.25315 | 5.8698 | 211.22 | 0.0321 | -24.3414 | -25.0367 |
|  | 161->167 | -0.34188 |  |  |  |  |  |
|  | 162->167 | 0.44924 |  |  |  |  |  |
| 24 | 158->167 | 0.58726 | 5.9643 | 207.88 | 0.0327 | 45.0466 | 48.1905 |
| 25 | 165->172 | 0.32161 | 6.0398 | 205.28 | 0.1595 | 123.8773 | 126.0261 |
|  | 165->173 | 0.30877 |  |  |  |  |  |
| 26 | 159->166 | -0.25114 | 6.0534 | 204.82 | 0.0426 | -31.7489 | -33.4821 |
|  | 165->172 | 0.46866 |  |  |  |  |  |
| 27 | 163->169 | -0.2485 | 6.0660 | 204.39 | 0.2244 | -10.0863 | -19.5487 |
|  | 165->172 | -0.37577 |  |  |  |  |  |
|  | 165->173 | 0.38321 |  |  |  |  |  |
| 28 | 159->167 | 0.27349 | 6.1033 | 203.14 | 0.2479 | -282.3155 | -297.6514 |
| 29 | 151->166 | -0.30152 | 6.1794 | 200.64 | 0.0078 | -6.9496 | -9.5729 |
|  | 154->166 | 0.3022 |  |  |  |  |  |
| 30 | 164->170 | 0.53399 | 6.2039 | 199.85 | 0.0393 | 46.0084 | 44.6198 |

Number of the excited states; Only transitions with contribution over 10.0% were listed; Configuration-interaction coefficient; Excitation energy; Wavelength; Rotatory strength in velocity form (10^-40^ cgs); Oscillator strength; Rotatory strength in length form (10^-40^ cgs).

1. Key transitions, oscillator strengths, and rotatory strengths in the ECD spectrum of conformer (12*S*28*S*31*R*)-**3**-2 at the B3lyp/6-31G(d) level of theory in MeOH with IEFPCM solvent model.

| Num | transition | CI-coeff | ΔE (eV) | λ (nm) | f | Rvel | Rlen |
| --- | --- | --- | --- | --- | --- | --- | --- |
| 1 | 164->166 | 0.62573 | 3.4962 | 354.63 | 0.0957 | -24.5425 | -38.5389 |
|  | 165->166 | 0.26268 |  |  |  |  |  |
| 2 | 165->167 | 0.63862 | 3.8360 | 323.21 | 0.5550 | -13.2899 | -11.0805 |
| 3 | 156->166 | 0.40981 | 4.0832 | 303.65 | 0.0033 | 15.2843 | 16.1822 |
|  | 157->166 | 0.29165 |  |  |  |  |  |
|  | 165->166 | 0.27375 |  |  |  |  |  |
| 4 | 165->166 | 0.57335 | 4.0968 | 302.64 | 0.0122 | 15.8799 | 17.0821 |
| 5 | 164->167 | 0.66253 | 4.3849 | 282.75 | 0.0259 | 13.0447 | 11.7935 |
| 6 | 160->166 | -0.27576 | 4.6105 | 268.91 | 0.0877 | -31.0219 | -25.2241 |
|  | 161->166 | 0.38584 |  |  |  |  |  |
|  | 164->168 | -0.38225 |  |  |  |  |  |
| 7 | 163->167 | -0.3418 | 4.8005 | 258.27 | 0.0225 | -30.8891 | -27.0137 |
|  | 165->169 | 0.5123 |  |  |  |  |  |
| 8 | 163->167 | 0.35316 | 4.8728 | 254.44 | 0.0874 | 131.37 | 137.3357 |
|  | 163->169 | 0.28189 |  |  |  |  |  |
|  | 165->169 | 0.30898 |  |  |  |  |  |
| 9 | 163->166 | 0.55398 | 4.9383 | 251.07 | 0.0489 | -9.4081 | -11.2125 |
|  | 164->168 | 0.34817 |  |  |  |  |  |
| 10 | 161->166 | -0.29855 | 4.9891 | 248.51 | 0.1066 | -113.2547 | -117.501 |
|  | 163->166 | 0.37761 |  |  |  |  |  |
|  | 164->168 | -0.37001 |  |  |  |  |  |
| 11 | 153->167 | 0.266 | 5.0626 | 244.90 | 0.0165 | 23.6155 | 25.6379 |
|  | 155->167 | -0.26263 |  |  |  |  |  |
|  | 160->167 | -0.2508 |  |  |  |  |  |
| 12 | 160->166 | -0.32777 | 5.1966 | 238.59 | 0.0050 | -9.1732 | -9.1391 |
|  | 165->168 | 0.49121 |  |  |  |  |  |
| 13 | 162->166 | 0.6775 | 5.2592 | 235.75 | 0.0030 | 3.6454 | 4.4843 |
| 14 | 161->167 | 0.36465 | 5.2888 | 234.43 | 0.0071 | 8.0008 | 9.9166 |
|  | 163->169 | 0.31637 |  |  |  |  |  |
|  | 165->173 | -0.2421 |  |  |  |  |  |
| 15 | 161->167 | 0.35269 | 5.3423 | 232.08 | 0.0791 | 26.2582 | 28.2277 |
|  | 163->167 | 0.42028 |  |  |  |  |  |
|  | 163->169 | -0.24045 |  |  |  |  |  |
| 16 | 160->166 | 0.44949 | 5.4535 | 227.35 | 0.0087 | 14.5677 | 15.9774 |
|  | 161->166 | 0.33732 |  |  |  |  |  |
|  | 165->168 | 0.30591 |  |  |  |  |  |
| 17 | 155->167 | 0.30712 | 5.5217 | 224.54 | 0.0053 | -48.9842 | -50.4116 |
|  | 155->170 | -0.30366 |  |  |  |  |  |
|  | 155->171 | 0.2295 |  |  |  |  |  |
| 18 | 160->167 | 0.23865 | 5.6297 | 220.23 | 0.0498 | 36.8701 | 38.4425 |
|  | 164->169 | 0.45555 |  |  |  |  |  |
| 19 | 164->169 | 0.42542 | 5.7050 | 217.33 | 0.0122 | 19.8658 | 22.8574 |
|  | 165->170 | -0.36984 |  |  |  |  |  |
| 20 | 160->167 | 0.33608 | 5.7294 | 216.40 | 0.1481 | -35.195 | -41.0893 |
|  | 164->169 | -0.23562 |  |  |  |  |  |
|  | 165->170 | -0.30296 |  |  |  |  |  |
| 21 | 159->166 | -0.28325 | 5.7972 | 213.87 | 0.1611 | 98.6025 | 98.49 |
|  | 160->167 | 0.33917 |  |  |  |  |  |
|  | 161->167 | -0.26147 |  |  |  |  |  |
|  | 161->168 | 0.26189 |  |  |  |  |  |
| 22 | 154->166 | -0.25938 | 5.8712 | 211.17 | 0.1785 | -69.2146 | -73.7455 |
|  | 159->166 | 0.30931 |  |  |  |  |  |
|  | 161->168 | 0.32636 |  |  |  |  |  |
| 23 | 158->167 | 0.60747 | 5.9753 | 207.50 | 0.0290 | 24.766 | 27.1989 |
| 24 | 165->172 | 0.51824 | 6.0396 | 205.28 | 0.1450 | 96.9644 | 97.6675 |
| 25 | 162->167 | 0.57956 | 6.0516 | 204.88 | 0.0297 | -2.1413 | -2.7117 |
| 26 | 165->172 | 0.36803 | 6.0675 | 204.34 | 0.1672 | 117.3915 | 116.7509 |
|  | 165->173 | 0.4145 |  |  |  |  |  |
| 27 | 157->167 | 0.26429 | 6.0842 | 203.78 | 0.2040 | -306.5466 | -328.5664 |
|  | 164->175 | 0.23455 |  |  |  |  |  |
| 28 | 157->166 | -0.29697 | 6.1216 | 202.53 | 0.0303 | 29.9939 | 29.3678 |
|  | 159->166 | 0.41108 |  |  |  |  |  |
| 29 | 164->170 | 0.46974 | 6.1511 | 201.56 | 0.1053 | -52.4987 | -52.8416 |
| 30 | 151->166 | -0.27966 | 6.2642 | 197.92 | 0.0055 | 8.497 | 6.6911 |
|  | 154->166 | 0.35812 |  |  |  |  |  |

Number of the excited states; Only transitions with contribution over 10.0% were listed; Configuration-interaction coefficient; Excitation energy; Wavelength; Rotatory strength in velocity form (10^-40^ cgs); Oscillator strength; Rotatory strength in length form (10^-40^ cgs).

1. Key transitions, oscillator strengths, and rotatory strengths in the ECD spectrum of conformer (12*S*28*S*31*R*)-**3**-3 at the B3lyp/6-31G(d) level of theory in MeOH with IEFPCM solvent model.

| Num | transition | CI-coeff | ΔE (eV) | λ (nm) | f | Rvel | Rlen |
| --- | --- | --- | --- | --- | --- | --- | --- |
| 1 | 164->166 | 0.66215 | 3.5307 | 351.16 | 0.1894 | -25.2875 | -38.5805 |
| 2 | 165->166 | -0.31009 | 3.9632 | 312.84 | 0.3197 | 71.8161 | 76.1316 |
|  | 165->167 | 0.58394 |  |  |  |  |  |
| 3 | 156->166 | 0.36474 | 4.0564 | 305.65 | 0.0092 | 33.3053 | 36.2499 |
|  | 157->166 | 0.39825 |  |  |  |  |  |
| 4 | 160->166 | -0.27386 | 4.5020 | 275.40 | 0.0667 | 39.9943 | 45.1367 |
|  | 164->167 | 0.49443 |  |  |  |  |  |
|  | 164->168 | -0.25608 |  |  |  |  |  |
| 5 | 165->166 | 0.58813 | 4.5361 | 273.33 | 0.0278 | -6.2768 | -7.094 |
|  | 165->167 | 0.33078 |  |  |  |  |  |
| 6 | 160->166 | 0.32475 | 4.7609 | 260.42 | 0.0943 | -49.1693 | -55.2362 |
|  | 162->166 | -0.2497 |  |  |  |  |  |
|  | 164->167 | 0.41779 |  |  |  |  |  |
| 7 | 165->168 | -0.27245 | 4.8572 | 255.26 | 0.0850 | -33.4542 | -26.4043 |
|  | 165->169 | 0.5618 |  |  |  |  |  |
| 8 | 161->167 | -0.26745 | 4.9518 | 250.38 | 0.1796 | -75.4847 | -77.8378 |
|  | 163->167 | -0.29043 |  |  |  |  |  |
|  | 163->169 | 0.35533 |  |  |  |  |  |
|  | 165->173 | -0.23203 |  |  |  |  |  |
| 9 | 160->166 | 0.24878 | 5.0488 | 245.57 | 0.0102 | -1.945 | -1.6235 |
|  | 162->166 | 0.58899 |  |  |  |  |  |
| 10 | 152->167 | 0.31654 | 5.1199 | 242.16 | 0.0719 | 18.1419 | 21.1231 |
|  | 154->167 | 0.25241 |  |  |  |  |  |
|  | 155->167 | -0.23397 |  |  |  |  |  |
| 11 | 161->166 | -0.22482 | 5.1851 | 239.12 | 0.0211 | -32.205 | -25.0273 |
|  | 161->167 | 0.40126 |  |  |  |  |  |
| 12 | 164->168 | 0.50338 | 5.2260 | 237.24 | 0.0767 | -9.9589 | -13.5736 |
|  | 164->169 | 0.23759 |  |  |  |  |  |
| 13 | 163->166 | -0.38603 | 5.2752 | 235.03 | 0.0364 | -66.311 | -65.5308 |
|  | 163->167 | 0.41141 |  |  |  |  |  |
|  | 163->169 | 0.28772 |  |  |  |  |  |
| 14 | 158->166 | 0.46955 | 5.5006 | 225.40 | 0.0378 | 4.7955 | 1.1662 |
|  | 161->167 | 0.22507 |  |  |  |  |  |
| 15 | 155->170 | -0.24554 | 5.5303 | 224.19 | 0.0389 | -60.1694 | -67.0385 |
|  | 158->166 | 0.248 |  |  |  |  |  |
| 16 | 163->166 | 0.44546 | 5.5814 | 222.14 | 0.0127 | -11.6134 | -11.6145 |
|  | 163->167 | 0.33911 |  |  |  |  |  |
| 17 | 160->167 | 0.31787 | 5.6241 | 220.45 | 0.1584 | 114.5448 | 116.9588 |
| 18 | 158->166 | 0.26683 | 5.7041 | 217.36 | 0.0019 | -8.6547 | -10.0779 |
|  | 163->166 | 0.2855 |  |  |  |  |  |
|  | 165->168 | 0.2984 |  |  |  |  |  |
| 19 | 159->166 | 0.3094 | 5.7432 | 215.88 | 0.0662 | -87.2303 | -91.0054 |
|  | 165->168 | 0.38803 |  |  |  |  |  |
| 20 | 158->166 | 0.23591 | 5.7914 | 214.08 | 0.0461 | 29.0775 | 26.8946 |
|  | 159->166 | 0.23262 |  |  |  |  |  |
|  | 161->166 | -0.22846 |  |  |  |  |  |
| 21 | 165->170 | 0.38468 | 5.9330 | 208.98 | 0.0011 | 14.0055 | 15.8537 |
| 22 | 153->166 | -0.27213 | 5.9811 | 207.29 | 0.0249 | -73.2128 | -75.3214 |
|  | 157->166 | 0.35725 |  |  |  |  |  |
|  | 159->166 | 0.31321 |  |  |  |  |  |
| 23 | 153->166 | 0.34493 | 6.0283 | 205.67 | 0.1417 | -26.2475 | -29.1572 |
|  | 162->167 | 0.2282 |  |  |  |  |  |
| 24 | 160->167 | 0.30209 | 6.0433 | 205.16 | 0.2596 | -122.8483 | -130.4331 |
|  | 160->168 | 0.27307 |  |  |  |  |  |
|  | 162->168 | -0.24021 |  |  |  |  |  |
| 25 | 165->171 | 0.45601 | 6.1125 | 202.84 | 0.2164 | 128.9707 | 134.4507 |
|  | 165->172 | -0.2623 |  |  |  |  |  |
|  | 165->173 | -0.23292 |  |  |  |  |  |
| 26 | 162->167 | 0.38869 | 6.1274 | 202.34 | 0.0887 | 37.5916 | 31.7333 |
|  | 165->173 | 0.25322 |  |  |  |  |  |
| 27 | 165->171 | 0.24693 | 6.1629 | 201.18 | 0.3073 | 8.2424 | 4.5189 |
|  | 165->172 | -0.26453 |  |  |  |  |  |
|  | 165->173 | 0.38237 |  |  |  |  |  |
| 28 | 158->167 | 0.41855 | 6.2137 | 199.53 | 0.0893 | -42.022 | -44.3717 |
|  | 161->169 | -0.33458 |  |  |  |  |  |
| 29 | 158->167 | 0.3329 | 6.2204 | 199.32 | 0.1214 | 39.2815 | 39.9059 |
|  | 159->167 | -0.23975 |  |  |  |  |  |
|  | 161->169 | 0.25207 |  |  |  |  |  |
| 30 | 151->166 | 0.26967 | 6.2237 | 199.21 | 0.1011 | 0.11 | -3.2472 |
|  | 161->169 | -0.23688 |  |  |  |  |  |
|  | 162->167 | 0.24385 |  |  |  |  |  |

Number of the excited states; Only transitions with contribution over 10.0% were listed; Configuration-interaction coefficient; Excitation energy; Wavelength; Rotatory strength in velocity form (10^-40^ cgs); Oscillator strength; Rotatory strength in length form (10^-40^ cgs).

1. Key transitions, oscillator strengths, and rotatory strengths in the ECD spectrum of conformer (12*S*28*S*31*R*)-**3**-4 at the B3lyp/6-31G(d) level of theory in MeOH with IEFPCM solvent model.

| Num | transition | CI-coeff | ΔE (eV) | λ (nm) | f | Rvel | Rlen |
| --- | --- | --- | --- | --- | --- | --- | --- |
| 1 | 164->166 | 0.59231 | 3.4776 | 356.52 | 0.0536 | -28.7996 | -41.1632 |
|  | 165->166 | 0.30671 |  |  |  |  |  |
| 2 | 164->166 | 0.32297 | 3.7550 | 330.18 | 0.5219 | 10.2738 | 14.2927 |
|  | 165->166 | -0.38946 |  |  |  |  |  |
|  | 165->167 | 0.4704 |  |  |  |  |  |
| 3 | 165->166 | 0.49264 | 3.9436 | 314.40 | 0.0947 | 46.8284 | 47.592 |
|  | 165->167 | 0.46577 |  |  |  |  |  |
| 4 | 156->166 | 0.4398 | 4.0864 | 303.41 | 0.0074 | 29.5764 | 32.137 |
|  | 157->166 | 0.26655 |  |  |  |  |  |
|  | 162->166 | 0.25846 |  |  |  |  |  |
| 5 | 164->167 | 0.67111 | 4.3288 | 286.42 | 0.0267 | 6.9774 | 6.0711 |
| 6 | 161->166 | -0.24515 | 4.5895 | 270.15 | 0.0965 | -96.9508 | -93.1119 |
|  | 162->166 | 0.38015 |  |  |  |  |  |
|  | 164->168 | -0.34766 |  |  |  |  |  |
| 7 | 163->166 | 0.3065 | 4.7413 | 261.50 | 0.0187 | -21.3099 | -18.0308 |
|  | 163->167 | -0.24599 |  |  |  |  |  |
|  | 165->169 | 0.52707 |  |  |  |  |  |
| 8 | 163->166 | 0.45473 | 4.7854 | 259.09 | 0.0888 | 64.4144 | 69.1913 |
|  | 165->169 | -0.37406 |  |  |  |  |  |
| 9 | 162->166 | 0.27867 | 4.9668 | 249.63 | 0.1083 | -20.6871 | -22.3891 |
|  | 164->168 | 0.52972 |  |  |  |  |  |
| 10 | 163->166 | 0.35316 | 4.9904 | 248.45 | 0.0652 | -50.7842 | -56.32 |
|  | 163->167 | 0.37658 |  |  |  |  |  |
| 11 | 160->166 | 0.24658 | 5.0495 | 245.54 | 0.0004 | -0.5415 | 0.3324 |
|  | 165->168 | 0.46882 |  |  |  |  |  |
| 12 | 165->168 | 0.43159 | 5.0868 | 243.74 | 0.0305 | 23.3869 | 24.827 |
| 13 | 161->166 | 0.57463 | 5.2799 | 234.82 | 0.0048 | 10.1319 | 10.2846 |
|  | 162->166 | 0.27312 |  |  |  |  |  |
| 14 | 163->167 | 0.41441 | 5.3002 | 233.92 | 0.0394 | -7.7665 | -4.1695 |
|  | 163->169 | -0.36346 |  |  |  |  |  |
|  | 165->171 | 0.25264 |  |  |  |  |  |
| 15 | 160->166 | 0.30542 | 5.3253 | 232.82 | 0.0193 | 21.7808 | 19.1553 |
|  | 161->167 | -0.30162 |  |  |  |  |  |
|  | 162->166 | -0.27327 |  |  |  |  |  |
|  | 162->167 | 0.31806 |  |  |  |  |  |
| 16 | 160->166 | 0.46036 | 5.4558 | 227.25 | 0.0121 | 21.2969 | 21.6636 |
|  | 162->167 | -0.22386 |  |  |  |  |  |
| 17 | 164->169 | 0.55545 | 5.5324 | 224.10 | 0.0038 | 7.1347 | 5.8622 |
| 18 | 155->170 | 0.24187 | 5.5359 | 223.96 | 0.0223 | -56.2447 | -55.2901 |
|  | 164->169 | 0.28609 |  |  |  |  |  |
| 19 | 165->170 | 0.43194 | 5.6053 | 221.19 | 0.0119 | 42.5248 | 47.522 |
|  | 165->172 | -0.23947 |  |  |  |  |  |
| 20 | 160->167 | 0.25806 | 5.7106 | 217.11 | 0.3496 | 87.8222 | 83.2461 |
|  | 162->168 | 0.31896 |  |  |  |  |  |
| 21 | 160->167 | 0.42852 | 5.7547 | 215.45 | 0.0404 | -1.9558 | -1.8942 |
|  | 162->167 | 0.28891 |  |  |  |  |  |
| 22 | 154->166 | -0.26399 | 5.8345 | 212.50 | 0.1786 | -188.7577 | -195.0648 |
|  | 159->166 | 0.32854 |  |  |  |  |  |
|  | 162->168 | 0.30438 |  |  |  |  |  |
| 23 | 163->169 | 0.34108 | 5.8683 | 211.28 | 0.3621 | 146.2247 | 146.8907 |
|  | 165->171 | 0.51879 |  |  |  |  |  |
| 24 | 158->166 | -0.29701 | 5.9672 | 207.78 | 0.0012 | -0.4541 | -0.3331 |
|  | 158->167 | 0.50605 |  |  |  |  |  |
| 25 | 157->167 | -0.23894 | 6.0558 | 204.74 | 0.1470 | -76.3105 | -84.8379 |
|  | 161->167 | 0.29321 |  |  |  |  |  |
| 26 | 157->166 | -0.32431 | 6.1018 | 203.19 | 0.0015 | 11.3105 | 10.4833 |
|  | 159->166 | 0.33639 |  |  |  |  |  |
|  | 161->167 | 0.29117 |  |  |  |  |  |
| 27 | 159->166 | -0.24465 | 6.1162 | 202.71 | 0.0710 | -55.51 | -59.3445 |
|  | 161->167 | 0.26471 |  |  |  |  |  |
|  | 164->170 | 0.28122 |  |  |  |  |  |
| 28 | 164->170 | 0.42854 | 6.1597 | 201.28 | 0.0951 | -4.7066 | -4.7783 |
| 29 | 151->166 | -0.31024 | 6.2106 | 199.63 | 0.0005 | 5.1515 | 1.6303 |
|  | 154->166 | 0.38585 |  |  |  |  |  |
| 30 | 165->174 | 0.38743 | 6.2150 | 199.49 | 0.0700 | -42.7246 | -47.8829 |

Number of the excited states; Only transitions with contribution over 10.0% were listed; Configuration-interaction coefficient; Excitation energy; Wavelength; Rotatory strength in velocity form (10^-40^ cgs); Oscillator strength; Rotatory strength in length form (10^-40^ cgs).

Fermentation, Extraction, and Isolation

The fungal strain was grown on PDA slants at 25 °C for 7 days. Agar plugs were cut into small pieces (about 0.5 × 0.5 × 0.5 cm^3^) aseptically. Four Erlenmeyer flasks (1 L), each containing 250 mL of PDA media, were inoculated with 100 agar pieces. The seed culture inoculated was prepared at 28 °C on a rotary shaker at 180 rpm for three days. 200 Fernbach flasks (1 L), each containing 105 g of rice, 45 g of millet, and 10 mL of seed culture, were used for static fermentation. After 30 days, the solid fermentation was dried, smashed, and extracted with EtOAc. The EtOAc extract (80 g) was obtained after removing the organic solvent via evaporation under reduced pressure.

Loaded to silica gel (PE/EtOAc, *v/v*, 100:0→0:100 then EtOAc/MeOH, *v/v*, 100:0→0:100), the extract was chromatographed into 12 fractions, label as FR1–FR12. FR6 was resolved using Sephadex LH-20 to produce compound **7** (0.9 mg). FR7 was subjected to Sephadex LH-20 and RP-HPLC (20% to 80% MeCN in H_2_O, 15 min → 80% to 100% MeCN in H_2_O, 5 min; flow rate 10 mL/min) to give compound **8** (6.3 mg, *t*_R_ 19.8 min) and compound **9** (5.0 mg, *t*_R_ 15.7 min). FR8 was purified by Sephadex LH-20 (methanol) and RP-HPLC (20% to 80% MeCN in H_2_O, 20 min → 80% to 100% MeCN in H_2_O, 5 min → 100% MeCN in H_2_O, 10 min; flow rate 10 mL/min) to give compound **1** (2.4 mg, *t*_R_ 29.3 min). FR9 was loaded to Sephadex LH-20 (methanol) and further separated by RP-HPLC (70% to 100% MeCN in H_2_O, 20 min; flow rate 10 mL/min) to give compound **2** (0.8 mg, *t*_R_ 16.7 min) and **3** (0.8 mg, *t*_R_ 15.4 min). FR10 was chromatographed on Sephadex LH-20 (methanol) and further purified by preparative HPLC (20% to 80% MeCN in H_2_O, 20 min → 80% to 100% MeCN in H_2_O, 5 min) to give compound **4** (2.0 mg, *t*_R_ 19.8 min), compound **5** (2.2 mg, *t*_R_ 20.9 min) and compound **6** (2.4 mg, *t*_R_ 15.9 min). FR12 was purified by Sephadex LH-20 and RP-HPLC (20% to 80% MeCN in H_2_O, 15 min → 80% to 100% MeCN in H_2_O, 5 min) to give compound **10** (7.0 mg, *t*_R_ 15.1 min). FR13 was chromatographed by Sephadex LH-20 and further purified by preparative HPLC (20% to 80% MeCN in H_2_O, 20 min → 80% to 100% MeCN in H_2_O, 5 min) to give compound **11** (0.9 mg, *t*_R_ 16.8 min).

NMR Calculation

The conformer rotamer ensemble sampling tool (crest) [^1^](#_ENREF_1) was utilized to generate candidate conformers, which were then subjected to DFT calculations using Gaussian 16 program [^2^](#_ENREF_2). The conformers for compounds **1** and **2** were optimized at B3lyp/6-31G(d) level of theory with Grimme's D3 dispersion correction. The energy window was set to 10 kcal/mol. After that, the electronic energies of the optimized conformations were calculated at M062X/6-311+G(2d,p). Those conformers with a population over 2% were subjected to subsequent calculations. For simplified conformers of compound **3**, electronic energy was obtained at the M06-2X/ma-TZVP level of theory, and thermal correction to Gibbs free energy was obtained at the M06-2X/def2-TZVP level of theory.

The shielding constants, calculated by the GIAO method at mPW1PW91/6-31+G(d,p), were converted into chemical shifts. TMS at 0 ppm (*δ*_cal_ = *σ*_TMS_ – *σ*_cal_) was used as the reference, where the *σ*_TMS_ was the shielding constant of TMS calculated at the same level [^3^](#_ENREF_3). The DP4+ probabilities of each possible candidate were calculated with the EXCEL spreadsheet provided by Sarotti *et al* [^4^](#_ENREF_4). For each possible candidate, the parameters *a* and *b* of the linear regression *δ*_cal_ = *aδ*_exp_ + *b*; the correlation coefficient, *R*^2^; the mean absolute error (MAE) defined as Σn|*δ*_cal_ - *δ*_exp_|/n; the corrected mean absolute error, CMAE, defined as Σn|*δ*_corr_ - *δ*_exp_|/n, where *δ*_corr_ = (*δ*_cal_ - b)/a, were calculated [^5-7^](#_ENREF_5).

**References:**

1. Pracht, P.; Bohle, F.; Grimme, S., Automated exploration of the low-energy chemical space with fast quantum chemical methods. *Physical chemistry chemical physics : PCCP* **2020,** 22, (14), 7169-7192.

2. Frisch, M. J.; Trucks, G. W.; Schlegel, H. B.; Scuseria, G. E.; Robb, M. A.; Cheeseman, J. R.; Scalmani, G.; Barone, V.; Petersson, G. A.; Nakatsuji, H.; Li, X.; Caricato, M.; Marenich, A. V.; Bloino, J.; Janesko, B. G.; Gomperts, R.; Mennucci, B.; Hratchian, H. P.; Ortiz, J. V.; Izmaylov, A. F.; Sonnenberg, J. L.; Williams; Ding, F.; Lipparini, F.; Egidi, F.; Goings, J.; Peng, B.; Petrone, A.; Henderson, T.; Ranasinghe, D.; Zakrzewski, V. G.; Gao, J.; Rega, N.; Zheng, G.; Liang, W.; Hada, M.; Ehara, M.; Toyota, K.; Fukuda, R.; Hasegawa, J.; Ishida, M.; Nakajima, T.; Honda, Y.; Kitao, O.; Nakai, H.; Vreven, T.; Throssell, K.; Montgomery Jr., J. A.; Peralta, J. E.; Ogliaro, F.; Bearpark, M. J.; Heyd, J. J.; Brothers, E. N.; Kudin, K. N.; Staroverov, V. N.; Keith, T. A.; Kobayashi, R.; Normand, J.; Raghavachari, K.; Rendell, A. P.; Burant, J. C.; Iyengar, S. S.; Tomasi, J.; Cossi, M.; Millam, J. M.; Klene, M.; Adamo, C.; Cammi, R.; Ochterski, J. W.; Martin, R. L.; Morokuma, K.; Farkas, O.; Foresman, J. B.; Fox, D. J. *Gaussian 16 Rev. C.01*, Wallingford, CT, 2016.

3. Willoughby, P. H.; Jansma, M. J.; Hoye, T. R., A guide to small-molecule structure assignment through computation of (^1^H and ^13^C) NMR chemical shifts. *Nature protocols* **2014,** 9, (3), 643-60.

4. Grimblat, N.; Zanardi, M. M.; Sarotti, A. M., Beyond DP4: an improved probability for the stereochemical assignment of isomeric compounds using quantum chemical calculations of NMR shifts. *The Journal of organic chemistry* **2015,** 80, (24), 12526-34.

5. Wang, W.; Yang, J.; Liao, Y. Y.; Cheng, G.; Chen, J.; Mo, S.; Yuan, L.; Cheng, X. D.; Qin, J. J.; Shao, Z., Aspeterreurone A, a cytotoxic dihydrobenzofuran-phenyl acrylate hybrid from the deep-sea-derived fungus *Aspergillus terreus* CC-S06-18. *Journal of natural products* **2020,** 83, (6), 1998-2003.

6. Wang, W.; Yang, J.; Liao, Y. Y.; Cheng, G.; Chen, J.; Cheng, X. D.; Qin, J. J.; Shao, Z., Cytotoxic nitrogenated azaphilones from the deep-sea-derived fungus *Chaetomium globosum* MP4-S01-7. *Journal of natural products* **2020,** 83, (4), 1157-1166.

7. Wang, W.; Chen, R.; Luo, Z.; Wang, W.; Chen, J., Antimicrobial activity and molecular docking studies of a novel anthraquinone from a marine-derived fungus *Aspergillus versicolor*. *Natural product research* **2018,** 32, (5), 558-563.
